# Bioprocessing of *Talaromyces purpurogenus* for production of dihydromonascorubrin with a powerful antioxidant and antimicrobial activities integrated with the network-guided target prediction and molecular docking analyses

**DOI:** 10.1186/s12866-026-05707-z

**Published:** 2026-10-22

**Authors:** Ashraf S. A. El‑Sayed, Samar A. Khashana, Sobhy S. Abdel-Fatah, Abeer Eldeghidy, Nouran A. Abd El-Hady, Ashraf F. El-Baz, Manal A. El-Shal, Eman Fikry, Rawia A. Zayed, Wafaa M. Ismaeil

**Affiliations:** 1 Enzymology and Fungal Biotechnology Lab, Botany and Microbiology Department, Faculty of Science, Zagazig University, Zagazig, 44519 Egypt; 2 Drug Radiation Research Department, National Center for Radiation Research and Technology, Egyptian Atomic Energy Authority, Cairo, Egypt; 3 Botany and Microbiology Department, Faculty of Science, Mansoura University, Mansoura, Egypt; 4 Biochemistry Department, Faculty of Agriculture, Zagazig University, Zagazig, 44519 Egypt; 5 Department of Industrial Biotechnology, Faculty of Biotechnology, University of Sadat City, Sadat City, Egypt; 6 Department of Microbiology, Faculty of Applied Health Sciences Technology, Horus University New Damietta, New Damietta, Egypt; 7 Pharmacognosy Department, Faculty of Pharmacy, Zagazig University, Zagazig, Egypt

**Keywords:** *Talaromyces purpurogenus*, Pigments, Dihydromonascorubrin, Antimicrobial activity, γ- irradiation

## Abstract

The antibiotic resistance of pathogenic microorganisms due to the irresponsible use in healthcare and agricultural practices is one of the global threats for the human, especially immune-compromised patients. Thus, searching for a novel compounds from fungi with unique affinity to combat the drug-resistant microorganisms was the objective. *Talaromyces purpurogenus* # PZ292970.1, an endophyte of palm tree *Elaeis guineensis*, has the highest productivity of azaphilone pigment (25.5 AU410/ml), with a strong activity against *E. coli* (MIC value 6.8 µg/ml), *S. aureus* and *C. albicans* (MIC value 12.5 µg/ml). The extracted pigment of *T. purpurogenus* has a strong antibiofilm activity for *S. aureus* (86.2%), *C. albicans* (85.8%) and *E. coli* (79.7%), compared to the control. The TLC purified-pigment of *T. purpurogenus* had a maximum absorbance at λ_410_ nm, with a small one at λ_510_ nm, revealing the identity of the major peak as orange azaphilone pigment. The purified pigment gave a distinct single peak by HPLC, ensures their structural homogeneity. From the FT-IR analyses, the absorption and stretching vibration of the target compound functional groups ensures their spectral identity as polyketide azaphilone. The 1HNMR and 13CNMR spectra revealed characteristic proton signals for the aromatic polyketide related to dihydromonascorubrin derivatives. From LC–MS/MS, the purified compound had 385.2 m*/z* that were matched to the molecular mass of dihydromonascorubrin, compared the authentic dihydromonascorubrin (382.4 m*/z*) as suggest from the fragmentation pattern by CFM-ID software. The yield of azaphilone pigment was increased by 2 folds upon bioprocessing by Plackett–Burman design, and by 2.4 folds by γ-irradiation at 1.0 kGy. The pigment showed a powerful antioxidant activity with free radical scavenging and reducing properties as reported by DPPH, ABT and FRAP assays. Integrating target prediction and molecular docking in silico analyses, dihydromonascorubrin exhibited multi-target mechanisms linked to efflux-associated resistance in *E. coli* and peptidoglycan remodeling in *S. aureus.*

**Supplementary Information:**

The online version contains supplementary material available at https://doi.org/10.1186/s12866-026-05707-z.

## Introduction

The emerging of the antibiotic-resistant of pathogenic microorganisms is one of the vital threats to the global healthcare, mainly driven by the irresponsible use of antibiotics leading to an exponential increase of Multidrug-resistant bacteria (MDR) [1]. The misuse and overuse of antibiotics in animal, veterinary, and agriculture sectors, concomitantly contribute to the rapid spread of the antibiotic-resistance genes “Silent Pandemic” that could massively increase the human mortality rates [2]. The antimicrobial resistance is mostly attributed to the repercussions of overuse/irresponsible use of antibiotics predominantly in clinical treatment, agricultural practices, and animal healthcare [3–5]. So, in absence of the proper preventive measures, projections indicate that by 2050, the antimicrobial resistance (AMR) phenomena could potentially supersede all other causes of mortality worldwide [6]. Global estimates indicate that the direct fatalities linked to AMR predicted to be escalated to ~ 10 million deaths annually by 2050 [7]. The drug resistance mechanisms can be classified into intrinsic, acquired, and adaptive resistance [8, 9]. The intrinsic resistance is the universally shared features within the bacterial species, independent on antibacterial exposure, nor horizontal gene transfer (HGT) [10, 11]. The intrinsic resistance of Gram-negative bacteria is correlated to the alteration of the glycopeptide identity of their cell walls, preventing their permeability [12]. Acquired resistance refers to mutation or acquisition of new genetic materials from an external source via HGT, that makes the bacterial cells are more resistant to antibiotics [13–15]. The adaptive drug resistance is temporary, the bacterial cells to respond swiftly to the antibacterial challenges, but once the inducing signal is diminished, the cells were reverted to their original antibiotic susceptibility [16, 17]. Additionally, the enzymatic breakdown of the antibiotics is one of the common mechanisms to the antibiotic resistance [18, 19]. However, the biofilm formation on the bacterial surface that shields their cellular matrix, thus boost their viability has been recently recognized as the most common reason of drug-resistance [20]. The antimicrobial resistance has been considered as one of the greatest threats to human health worldwide, as documented by the Infectious Diseases Society of America (IDSA). Clinically, the patients infected with MDR bacteria are more vulnerable to severe consequences [21, 22], consequently, spreading of the antibacterial resistance halt the regular treatments of cancer patients, and organ-transplantation, and others [23]. Thus searching for novel antimicrobial compounds tackling the abnormal mechanisms of microbial drug resistance, is the ultimate objective.

Fungi are well known for their ability to produce a diverse array of secondary metabolites, terpenoids, flavonoids, polypeptides and meroterpenoids with various biological activities (reviewed by El-Sayed et al. [24–26]). Among these metabolites, fungal pigments were recognized as one of the prominent metabolites with miscellaneous activities like antioxidants, antimicrobials, anti-diabetes, antidepressants, anticancer and anti-cholesterols [27, 28]. Naturally, fungi are extraordinary sources for wide range of pigment production,carotenoids, melanins, azaphilones, flavins, phenazines, and quinones [29–32]. The chemical stability of the fungal pigment in response to light, temperature and pH are the major affordable criteria [33]. Several filamentous fungi have the ability to produce water-soluble value-added pigment such as anthraquinones, complex polyketides, and certain riboflavins, in addition to azaphilones like monascorubin and Rubropunctamine as reviewed by Lin et al. [34]. Among the well-known pigment-producing fungi, *Monoascus* species have generally accepted as food-grade pigment producer, however, the presence of citrinin is the major hurdle for commercial use of this genus [28]. Recently, *Talaromyces* species were reported to produce a considerable amounts of extracellular pigments of yellow, orange, red, dark violet, with diverse biological activities, with no signs of mycotoxin productivity [35]. Additionally, *Talaromyces* species were reported to produce rubratoxins, with anticancer activity by inhibiting protein phosphatases, suppressing cancer metastasis [36]. Microbial colorants have been traditionally used to increase the esthetic appeal of various products such as cosmetics, pharmaceuticals, food and textiles [37, 38]. Although the massive promising applications of pigments in therapeutic fields, the productivity, availability and the exploring their antimicrobial activities towards the drug resistant microorganisms remain the equivocal. Thus, the objective of this study was to isolate a novel fungal isolate with superior pigment productivity, maximizing their pigment productivity, assess their antimicrobial efficacy against MDR microorganisms, integrating the network pharmacology and molecular docking in silico analyses to explore their mode of antimicrobial action.

## Materials and methods

### Materials

The fruits of palm tree “*Elaeis guineensis”* inhabiting Aswan Botanical Garden, Aswan, were collected and used as source of fungal isolation. The plant was identified by Professor Rawia A. Zayed, Pharmacognosy Department, Faculty of Pharmacy, Zagazig University, and preserved as voucher specimen at the Pharmacognosy Department Herbarium, Faculty of Pharmacy, Zagazig University, with deposition # PDH-Eg26-1. The plant materials were collected according to the relevant institutional, national and international guidelines and legislations.

## Methods

### Isolation, identification, and screening for the pigment-producing endophytic fungi from *Elaeis guineensis*

The endophytic fungi inhabiting the fruits of the *E. guineensis* were isolated on potato dextrose agar (PDA) media with ampicillin (1 mg/l) [39]. The developed colonies were purified by subculturing on PDA, as slope and plate cultures, stored at 4 °C. The fungal cultures grown on PDA and Czapek’s-Dox agar media, incubated for 10 days at 28 °C, their macro- and microscopical features were daily scrutinized, and observed by Olympus BX51 microscope. The fungal isolates were identified to their species level based on their microscopic features as mycelium color, conidial heads, pigmentation, hyphal/mycelial identity, conidiophores branching patterns and conidial ontology [40–43].

The recovered endophytic fungal isolates were screened for their pigments production by grown on PDB [39, 44]. Briefly, two plugs of 7-days old of each fungal isolate were inoculated into 50 ml/250 mL Erlenmeyer conical flask of PDB, incubated at 30 °C for 10 days, and the cultures were filtered. Triplicates of each isolate were prepared. The whole culture were extracted with 70% ethanol (1/1 v/v), in continuous shaking for 1 h, then the mixtures were filtered, the extract was concentrated by rotary evaporator at 50 °C, till 5 ml [45, 46]. The UV–Vis spectra of the fungal extracts were analyzed at wavelength range 200–700 nm (RIGOL, ultra-3000 Series), compared to blank cultures extracts [47]. The fungal azaphilone pigments were quantified by the optical density at λ_410_ nm (yellow-orange pigments) and λ_510_ nm (red pigments) [48]. The pigments yield was expressed as AU410/mL for the yellow-orange pigments, and AU510/m for the red pigments [46].

The highest pigments-producing fungal isolates were molecularly identified based on their internal transcribed spacer (ITS) sequence [49]. The fungal genomic DNA (gDNA) was extracted by pulverizing the mycelia in liquid N_2_, then extracted by cetyltrimethyl-ammonium bromide (CTAB) buffer (2% CTAB, 2% PVP40, 0.2% 2 mercapto-ethanol, 20 mM EDTA, 1.4 M NaCl in 100 mM Tris–HCl, pH 8.0). The gDNA was used as a PCR template with the primers ITS4 5’-GGAAGTAAAAGTCGTAACAAGG-3’ and ITS5 5’-TCCTCCGCTTATT GATATGC-3’. The reaction mixture contains 2 × PCR master mixture (i-TaqTM, Cat.# 25,027), gDNA, primers (10 pmol/ml), completed to 20 ml with sterile distilled water, according to the kit manufacturer’s instructions. The PCR was programed to 94 °C for 2 min, denaturation at 94 °C for 30 s, annealing at 55 °C for 10 s, extension at 72 °C for 30 s for 35 cycles, and 72 °C for 2 min. The PCR amplicon was analyzed by 1.5% agarose gel in TBE buffer (Cat# AM9864), using 1kb DNA ladder (Cat. # PG010-55DI), and visualized by the gel documentation system (Vilber Lourmat, France). The amplicons were purified, sequenced, and the ITS sequences were non-redundantly BLAST searched on NCBI database. The phylogenetic analyses were constructed with Maximum Likelihood method by MEGA-X software package, with 1000 bootstrap replications [50].

### Antimicrobial and anti-biofilm assay of the extracted pigments

The potent fungal isolate “*T. purpurogenus”* was grown on PDB at standard conditions, as described above, the cultures were extracted with different solvent namely acetone, methylene chloride, methanol, methyl ethyl ketone, chloroform, hexane, and ethyl acetate in addition to acid precipitate. The extracts were concentrated by rotary evaporator, till oily residues, then dissolving in 1 methanol. The tested bacterial strains were *Klebsiella pneumonia, Pseudomonas aeruginosa, Escherichia coli* and *Enterobacter agglomerans* as a model of Gram-negative bacteria, *Staphylococcus aureus*, *S. epidermidis* and *Bacillus subtilis* as Gram-positive bacteria, in addition to pathogenic unicellular fungi as *Candida albicans* and *C. tropicalis,* were used to evaluate the antimicrobial activity of the *T. purpurogenus* extract. These bacterial isolates were recognized as multidrug resistant isolates, obtained from the intensive Care Unit of Zagazig University Hospital, Zagazig, Egypt. The bacterial inocula were adjusted at 0.5 McFarland (1–2) × 10^8^ CFU/ml, at λ600 nm. The yeast inocula were adjusted by 0.5 McFarland (1–5) × 10^6^ CFU/ml. The antimicrobial activity of the extracts was assessed by disc-diffusion assay, each disc was loaded with 10 μl (10 mg/ml) of each extract, placed on the surface the microbial plate culture, incubated for 24 h at 37 °C, and the diameter of the inhibition zones were measured by a Vernier caliper (mm) [44]. The biosafety instructions and precautions were strictly followed all over the experiment, the bacterial cultures isolates and remains of potential biological hazards were safely disposed via autoclaving. The minimum inhibitory concentrations (MIC) of the potent extracts of *T. purpurogenus* towards the different microbial isolates were assessed in Luria–Bertani (LB) broth. The tested microbial cultures in LB media were amended with different concentration of *T. purpurogenus* extract (from to 0 to 250.0 μg/ml), inoculated with bacteria at 5 × 10^7^ CFU/ml, and for *Candida* species at 2 × 10^7^ CFU/ml. The cultures were incubated at 37 °C for 48 h, and the cultural optical density was measured at λ_600_ nm with the ELISA plate technique [51].

The most susceptible microorganisms were used to assess their biofilm-forming capability in response to the extract of *T. purpurogenus,* compared to control cultures [52, 53]. Five mls of LB broth was poured into standard test tube inoculated with the tested bacterial isolates, in addition to *Candida s*pp with the methanolic extract of *T. purpurogenus* at their MIC values (12.5 µg/ml), and the cultures were incubated overnight at 37 °C [54]. The tubes were gently washed with distilled water to remove any unattached cells and media, then 500 μl of 0.1% crystal violet was added to each tube for 15 min, the tubes were photographed using a digital camera. For quantification of the crystal violet, 500 μl of acetic acid (30%) was added and the absorbance was measured at λ570 nm [55]. Three replications foe each treatment, and average of the absorbance compared to LB blank control was calculated. The biofilm inhibition percentage (R%) was expressed by the following,

$$\begin{aligned} \mathrm{R}{\%} &= \lambda_{570}\;\mathrm{nm}\;\mathrm{of}\;\mathrm{control}\\ &\quad -\;(\lambda_{570}\;\mathrm{nm}\;\mathrm{of}\;\mathrm{control}\\ &\quad -\;\lambda_{570}\;\mathrm{nm}\;\mathrm{of}\;\mathrm{sample})\times100 \end{aligned}$$

### UV-Vis, FT-IR, NMR and LC–MS/MS analyses of the orange azaphilone pigment from *T. purpurogenus*

*T. purpurogenus* was grown on PDB broth (Cat.# DF0549-17–9) for 10 days at 30 °C, then the cultures were filtered, and the filtrates were extracted with ethylacetate, the extract was concentrated, then dissolved in 5 ml methanol. The extract was scanned by UV–Vis at λ200-λ500 nm (RIGOL, Ultra-3000 Spectrophotometer) with methanol as a baseline. The crude extract was checked by analytical TLC (Silica gel 60 F254, Merck KGaA, Germany) with methylene chloride: methanol: water (6:4:1 v/v), and the colored spots of orange-azaphilone pigments was observed. The target fractions of orange-yellow azaphilone pigment was eluted by scraping off the silica gel spots containing the pigments, dissolving in methanol, and checking the recovery ratio of the target compound by TLC again at the same conditions. The purity of the extracted compound was analyzed by High Performance Liquid Chromatography (YOUNG In, Korea) of C18 column (Cat.# 959,963–902) with mobile phase acetonitrile/water with 0.1% formic acid (60:40 v/v) at flow rate 1.0 ml/min for 20 min, and the absorbance of the sample was measured at wavelength λ_410_ nm [56].

The InfraRed (FT-IR) spectra of the sample were determined using a JASCO FT-IR 3600 Spectrophotometer, sample was pulverized in KBr pellets, and the spectra were recorded from 4000 to 500 cm^−1^. The identity of purified compound was assessed by 1H-NMR and ^13^CNMR (JEOL, ECA-500II), the shifts and coupling constant (ppm) was determined.

The structure of purified compound was verified by LC–MS/MS (Thermo Scientific, LCQ Deca mass spectrometer, in a positive mode). The mobile phase was acetonitrile in 0.1% formic acid, and the samples were injected to a Hypersil Gold C18 column, with a gradient elution at 2–98% at flow rate 0.2 ml/min for 20 min. The chemical identity of the target compound was verified from their molecular mass. Furthermore the molecular fragmentation pattern of the selected peaks of the parent molecule, were analyzed by ESI–MS/MS. The molecular identity of the sample was verified from their molecular fragmentation pattern corresponding to the authentic one, as well as by Competitive Fragmentation modeling for metabolite identification software package (https://cfmid.wishartlab.com/).

### Bioprocessing of the azaphilone pigment by *T. purpurogenus* with Plackett–Burman design

The nutritional physicochemical parameters of *T. purpurogenus* were optimized to maximum their productivity of azaphilone by Plackett–Burman design [39, 57]. Eleven physicochemical parameters namely incubation period, shaking speed, pH, sucrose, glucose, fructose, yeast extract, malt extract, peptone, citric acid and sodium nitrate were optimized, as represented with high (+ 1) and low (−1) levels. The Plackett–Burman design follows the first order reaction as follows: $\mathrm{Y}\;=\;\upbeta 0\;+\;\sum\upbeta\mathrm{iXi}$.

Y is the yield of pigment, Xi is the independent variable, βi is the linear coefficient, and β0 is the intercept. The runs were conducted in three biological replicates, and the response was expressed by the means of compound yield at wavelength λ_410_ nm/ml.

### Effect of γ-irradiation of *T. purpurogenus* on their azaphilone pigment productivity

The effect of γ-irradiation of *T. purpurogenus* on their productivity of azaphilone was assessed. The fungal spore suspension was irradiated with different doses of γ-rays (0.25 to 2.5 kGy) using India’s ^60^Co-Gamma chamber 4000-A gamma irradiator at National Centre for Radiation Research and Technology, Egyptian Atomic Energy Authority, Cairo, Egypt. The irradiated fungal spores, in addition to the non-irradiated ones were cultured on sterilized PDB of pH 6.0, for 12 days at 30 °C, at standard conditions. After incubation the cultures were extracted with ethyl acetate, concentrated by rotary evaporator, and quantified by spectrophotometric assay at λ410 and λ510 nm [44, 58–60].

### Antioxidant properties of the purified azaphilone pigment of *T. purpurogenus*

The antioxidant properties of the purified azaphilone pigment of *T. purpurogenus* were determined by 1,1-diphenyl-2-picrylhydrazyl (DPPH) [61], Azinobis-benzo Thiazoline Sulphonic (ABTS) [62] free radical scavenging assays and Ferric reducing antioxidant power (FRAP) [63]. Briefly, the reaction mixture contains 500 μl of *T. purpurogenus* purified azaphilone pigment with 500 μl of DPPH in methanol, the mixture was incubated for 30 min in dark at room temperature, and the absorbance was measured at λ_517_ nm. DPPH solution with distilled water was used as control. Triplicate reactions were conducted, using ascorbic acid as a standard.

For ABTS assay, the sample (500 μl) was mixed with 500 μl of ABTS solution, incubated in dark for 15 min, then the absorbance was measured at λ_734_ nm, compared to Butyl-hydroxytoluene as standard solution. The scavenging activity was calculated at the inhibitory concentration IC_50_ for dose–response by the following equation:

$$\begin{aligned} &\%\;\mathrm{Free}\;\mathrm{radical}\;\mathrm{scavenging}\;\mathrm{activity}\\ &=\;\lbrack{\mathrm A}_{\mathrm{blank}}\;-{\mathrm A}_{\mathrm{sample}}\;\rbrack/{\mathrm A}_{\mathrm{blank}}\;\times\;100 \end{aligned}$$

A _sample_ is the sample absorbance, and A _blank_ is the absorbance of blank.

For ferric reducing antioxidant power (FRAP) assay, the sample (500 μl) was mixed with 500 μl of FRAP reagent, incubated in dark for 30 min at 37 °C, then the developed color was measured at λ_590_ nm, normalizing to ascorbic acid as standard solution.

### Network-guided target prediction, hub prioritization, and molecular docking analysis

#### Target fishing and organism-specific target prioritization

The antimicrobial activity of the purified dihydromonascorubrin was assessed by in silico analysis to identify the plausible microbial molecular targets using PharmMapper as the target-fishing platform (https://www.lilab-ecust.cn/pharmmapper/) [64, 65]. The retrieved hits were annotated using UniProt, and the non-microbial entries were excluded according to UniProt organism annotation. Bacterial and fungal hits were retained as the initial microbial target pool. Direct *Escherichia coli* and *Staphylococcus aureus* hits were retained as organism-specific candidates, while fungal target-family hits were searched against *Candida albicans*. For reliability, only reviewed UniProtKB/Swiss-Prot entries showing exact organism-specific target matches or direct components of the corresponding enzyme complex were retained in the final candidate microbial target lists.

#### Protein–protein interaction (PPI) network construction and topological analysis

PPI networks were constructed separately for each organism-specific target list using STRING database (ver.12.0; https://string-db.org/) [66]. The minimum interaction confidence score was set to 0.4. Disconnected nodes were hidden only for visualization when required, without excluding them from the original target lists. STRING interaction data were imported into Cytoscape (ver. 3.10.2,NIGMS, USA) [67], and topological parameters were calculated using the CytoNCA plugin. Betweenness centrality (BC), closeness centrality (CC) and degree centrality (DC) were used for hub prioritization. Hub selection was applied only to networks with sufficient connectivity. For analyzable networks, nodes with BC, CC, and DC values equal to or greater than their respective medians were retained as hub targets.

### Molecular docking analysis

#### Target selection and docking rationale

Molecular docking targets were selected through a network-guided and functionally informed prioritization strategy. For *E. coli*, the AcrAB-TolC efflux module was prioritized because it included highly ranked efflux-associated hubs within the core network. Since AcrA has been reported to lack a defined substrate-binding site, AcrB was selected as the ligand-binding representative of the AcrA-associated AcrAB-TolC module, together with TolC. For *S. aureus*, docking focused on the cell-wall remodeling/autolysin-associated module; accordingly, ATL was selected as the top-ranked hub, whereas LYTM was selected as an additional core hub representing peptidoglycan-associated peptidase activity. For *C. albicans*, LIP4 and LIP5 were selected because they represented the only connected, network-supported fungal targets.

#### Receptor and ligand preparation

The structures of the selected bacterial targets were retrieved from Protein Data Bank: *E. coli* TolC (PDB ID: 1EK9), *E. coli* AcrB (PDB ID: 4DX5), the *S. aureus* AtlA/AmiA catalytic amidase domain (PDB ID: 4KNL), and the *S. aureus* LytM catalytic domain (PDB ID: 4ZYB). The TolC trimeric biological assembly was retained, and because the selected structure lacks a co-crystallized ligand, the docking grid was centered on the Asp371/Asp374 constriction region, the reported narrow substrate-translocation gate of the TolC periplasmic entrance. For AcrB, ATL/AmiA, and LYTM, the corresponding ligand- or substrate analogue-bound structures were used to define the functional binding regions. Co-crystallized ligands or substrate analogues, water molecules, and non-essential heteroatoms were removed before docking, whereas the catalytic Zn^2^⁺ ion in LYTM was retained as part of the metallopeptidase active site. *C. albicans* LIP4 and LIP5 targets, AlphaFold-predicted models were used for exploratory model-based docking. The models corresponding to LIP4 (UniProt ID: Q9P8W1; AlphaFold ID: AF-Q9P8W1-F1) and LIP5 (UniProt ID: Q9P8W0; AlphaFold ID: AF-Q9P8W0-F1) were retrieved and inspected before receptor preparation. For both lipases, the predicted catalytic regions were guided by the conserved GYSGG motif, in which Ser194 represents the putative catalytic serine. For LIP5, the reported catalytic triad residues Ser194, Asp343, and His376 were also considered [68]. All receptors were prepared in UCSF Chimera v1.17.3 using the Dock Prep workflow, followed by conversion into PDBQT format using Open Babel v2.4.1. The 3D conformer of dihydromonascorubrin was obtained from PubChem [69], energy-minimized, and converted into PDBQT format using Open Babel.

#### Docking protocol

Docking was performed using AutoDock Vina v1.1.2. For all targets, exhaustiveness was set to 24, num modes to 20, and energy_range to 4 kcal/mol. The docking protocol was validated by redocking the co-crystallized ligands/substrate analogues of AcrB, AtlA/AmiA, and LytM. The generated poses were compared with their crystallographic conformations using DockRMSD [70], and minimum symmetry-corrected heavy-atom RMSD values ≤ 2.0 Å were considered acceptable [71]. The best-ranked pose was selected based on binding affinity and consistency with the predefined binding or catalytic region. Two-dimensional (2D) interaction diagrams and 3D binding poses were generated using Biovia Discovery Studio Visualizer (v21.1.0.20298). Docking results obtained from AlphaFold-predicted LIP4 and LIP5 models were interpreted as supportive exploratory evidence rather than crystal-structure-based confirmation.

### Statistical analysis

The experiments were conducted in three biological replicates, and the results were expressed by mean + SD. The significance was calculated by one-way ANOVA with Fisher’s Least Significant Difference of post hoc test.

## Results

### Isolation, screening for azaphilone pigment-producing endophytic fungi of *Elaeis guineensis*

**Fig. 1:**
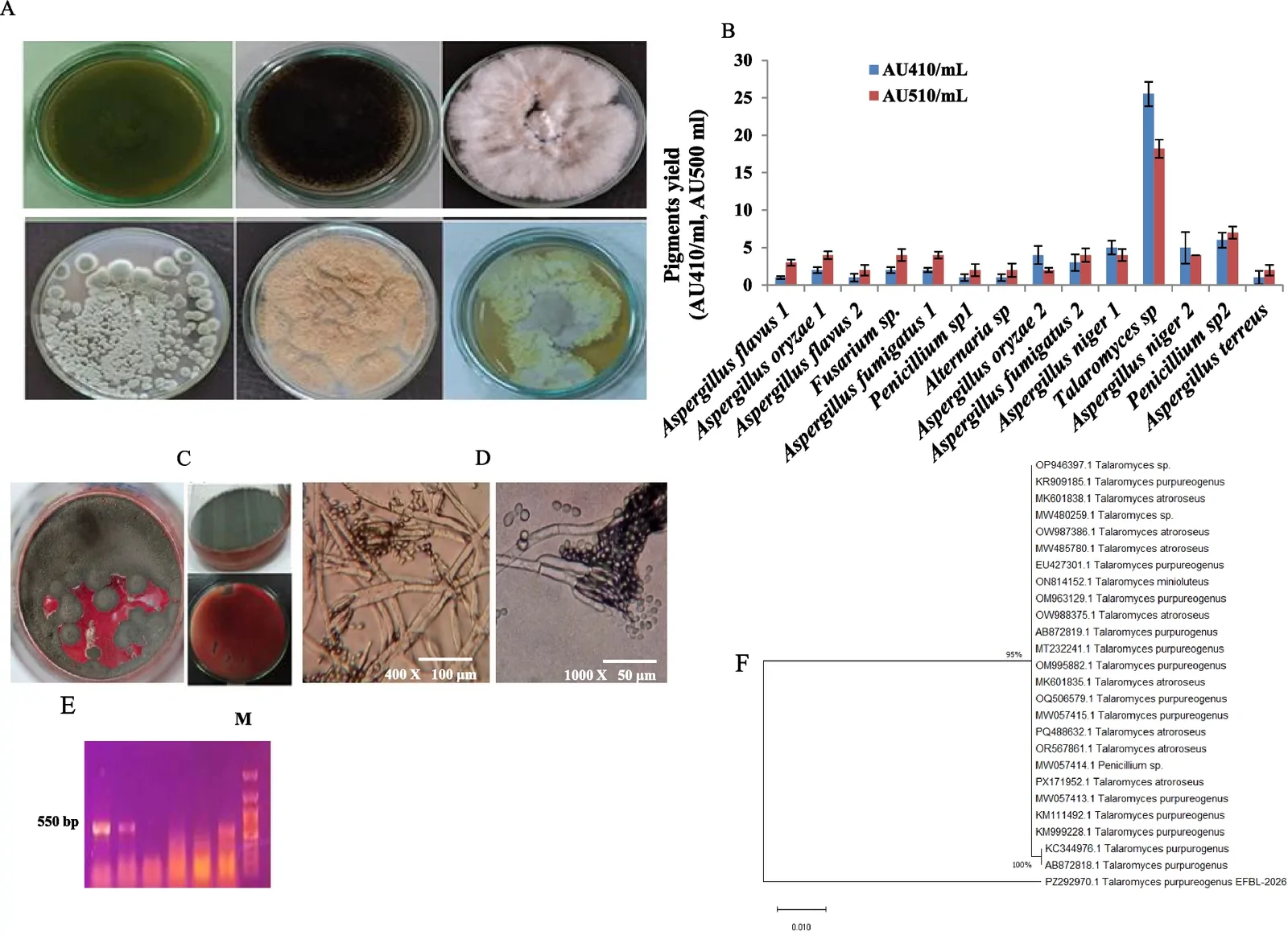
Screening, morphological and molecular identification of the potent pigment-producing fungal isolates. **A** Plate cultures of the recovered endophytic fungal isolates (1–6). **B** Extraction and quantification of the pigment from the selected fungal isolates. **C** Plate culture of the fungal isolate. **B** Microscopical features of the fungal isolate at 400 × and 1000 X. The PCR product of ITS region (**C**), and the phylogenetic analysis of the ITS sequence (**D**) by the Maximum Likelihood method with MEGA X software package

Fourteen endophytic fungal isolates of *E. guineensis* were isolated on PDA, morphologically identified based on their macro and microscopic features belong to five genera namely *Aspergillus*, *Fusarium, Penicillium, Alternaria* and *Talaromyces* (Table 1). These isolates were screened for their pigment-producing potency by growing on PDB medium, for 10 days, the cultures were extracted with 70% ethanol, and the pigment was dried and quantified as described above. The absorbance of the extracts was measured at wavelengths λ_410_ nm and λ_510_ nm, with the blank medium without fungal inocula as baseline. From the results (Table 1), the highest pigment-producing potency was reported for isolate #11 as quantified by 25.5 AU410/ml and 18.2 AU510/ml, followed by isolate #13 of 6.0 AU410/ml and 7.0 AU510/ml, respectively. Among the fourteen fungal isolates of *E. guineensis,* six isolates namely *A. flavus*, *A. oryzae*, *A. fumigatus*, *Penicillium* spp, and *Talaromyces* sp, as the representative of the pigments producing fungi were illustrated (Fig. 1). An obvious fluctuation on the productivity of pigments was recorded from the wavelengths 410 nm and 510 nm, for the other recovered fungal isolates.

**Table 1:** Isolation of the endophytic fungi from (stem and leaves) of *Elaeis guineensis*

Plant parts	**Isolate No**	**Fungal Isolate**	**AU410/mL**	**AU510/mL**
Stem	**1**	*Aspergillus flavus*_***1***_	**1.5 ± 0.2**	**3.1 ± 0.4**
	**2**	*Aspergillus oryzae*_*1*_	**2.2 ± 0.4**	**4.2 ± 0.5**
	**3**	*Aspergillus flavus*_***2***_	**1.7 ± 0.5**	**2.09 ± 0.4**
	**4**	*Fusarium* sp.	**2.8 ± 0.4**	**4.1 ± 0.5**
	**5**	*Aspergillus fumigatus*_*1*_	**2.7 ± 0.3**	**4.3 ± 0.5**
	**6**	*Penicillium* sp_1_	**1.7 ± 0.45**	**2.3 ± 0.7**
Leaves	**7**	*Alternaria* sp.	**1.4 ± 0.46**	**2.2 ± 0.8**
	**8**	*Aspergillus oryzae*2	**4.3 ± 1.2**	**2.1 ± 0.9**
	**9**	*Aspergillus fumigatus* 2	**3.2 ± 1.1**	**4.2 ± 1.1**
	**10**	*Aspergillus niger*_***1***_	**5 ± 0.9**	**4.1 ± 0.9**
	**11**	*Talaromyces* sp	**25.5 ± 2.6**	**18.2 ± 2.4**
	**12**	*Aspergillus niger*_***2***_	**5.1 ± 2.1**	**4.2 ± 0.8**
	**13**	*Penicillium* sp_2_	**6.1 ± 1.0**	**7.2 ± 1.1**
	**14**	*Aspergillus terreus*	**1.2 ± 0.9**	**21 ± 0.8**

The most potent pigment producing fungal isolate #11 “*Talaromyces* sp” was morphologically identified based on their microscopical features. The isolate has inability to grow below 20 °C on Czapek’s-Dox agar media, with highest growth at 30 °C, producing orange-red diffusing pigments. The fungal colonies have dark green conidia, yellow pigmented and encrusted aerial hyphae, yellow colony reverses, with orange/red to purplish red shades (Fig. 1). The isolate has a velvety texture, conidiophores strictly biverticillate, subterminal branches absent, metulae in verticils, conidia smooth and ellipsoidal. The colonies are moderately deep, very narrow margins, white-red mycelium, texture floccose, sparse sporulation, conidia are dull green, reverse coloration light yellow to brown. The soft ascomatal walls are comprised of multiple layers of interwoven hyphae and the ascomata mature quickly, within a few weeks, with an obvious diffusible red azaphilone “*Monascus* pigments”*.* The morphological descriptions of the current isolate typically follow those of *Talaromyces purpurogenus *(Stoll) Samson, Yilmaz, Frisvad & Seifert [42, 72]. *Talaromyces* with its ability to form soft yellow ascomata of interwoven hyphae, was formerly classified under *Penicillium* subg. *Biverticillium,* and recently identified as *Talaromyces* [72, 73].

The identity of the potent fungal isolate “*T. purpurogenus* sp*”* was further identified based on their ITS sequence, of their gDNA as a PCR template. The PCR amplicons were resolved as 550 bp on 1.5% agarose gel (Fig. 1E). The ITS sequence was non-redundantly BLAST searched on the genbank of NCBI database, with 100% similarity with those of *T. purpurogenus.* The ITS sequence of *T. purpurogenus* EFBL-2026 has been deposited to the genbank with accession # PZ292970.1, and their phylogenetic relatedness with the database deposited sequences was constructed by the Maximum likelihood by using MEGAX software package (Fig. 1). The phylogenetic analysis revealed that the isolate EFBL-2026 (PZ292970.1) clustered within the *T. purpurogenus* clade, showing a close phylogenetic relationship with authenticated *T. purpurogenus* strains, as strongly strongly supported by a bootstrap value of 100%, while the broader *Talaromyces* lineage received 95% bootstrap support. The scale bar indicates 0.010 nucleotide substitutions per site. The ITS sequence of the current isolate had a 99% similarity with those of *T. purpurogenus* KC344976, AB872818, KM111492, KR909185, KM999228, OM995882, OR567861, MT232241 and AB872819, with query coverage 100% and zero E-value. Collectively, from morphological features and molecular analysis of ITS sequence, the isolate was verified as the *T. purpurogenus.*

### Extraction, antimicrobial and anti-biofilm activity of the azaphilone pigments with the multi-drug resistant bacteria

**Fig. 2:**
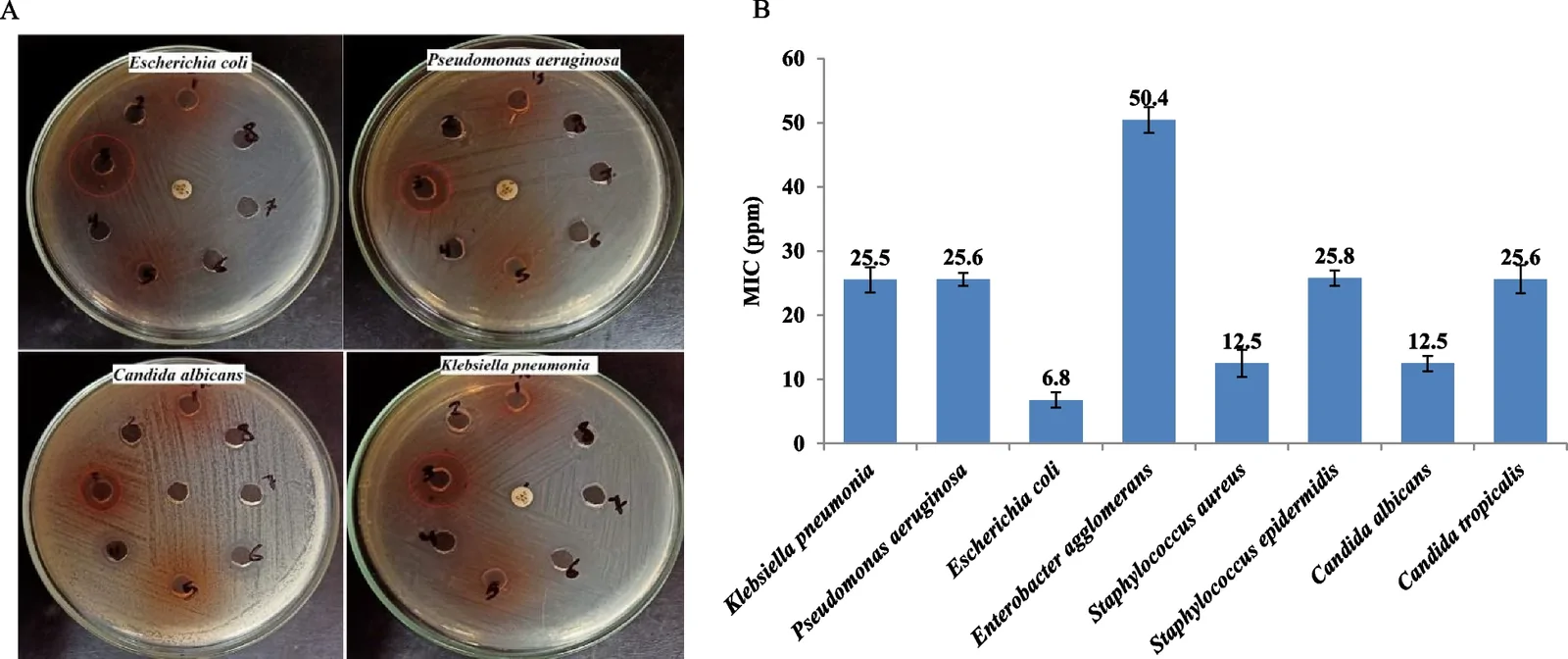
Biological activity guided-assay of the different extracts of *T. purpurogenus.* The 10 days old cultures of *T. purpurogenus* was extracted with acetone (1), methylene chloride (2), methanol (3), methyl ethyl ketone (4), chloroform (5), hexane (6), ethyl acetate (7) and acid precipitate (8). **A** Diameter of the inhibition zones of the tested microbial isolates in response to the different extracts of *T. purpurogenus*. **B** The minimum inhibitory concentration (MIC) of the methanolic extracts of the pigment towards the different microbial isolates

**Fig. 3:**
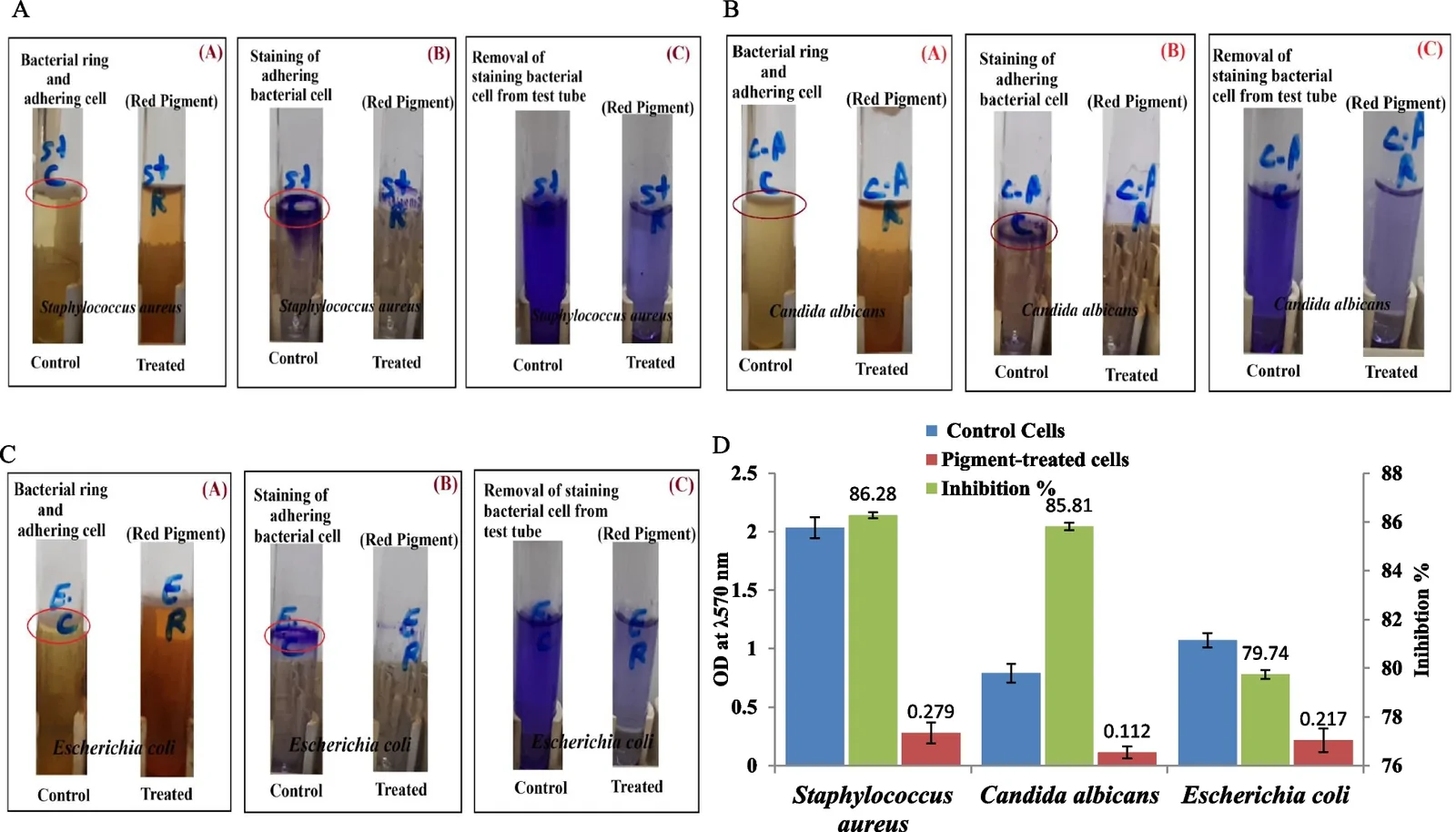
The antibiofilm activity of the extracted pigment of *T. purpurogenus* against *Staphylococcus aureus, Candida albicans*, and *E. coli*. The development of biofilm *S. aureus* (**A**), *C. albicans* (**B**), and *E. coli* (**C**) in response to the presence of extracted pigment compared to the control, by crystal violet stain. **D** The antibiofilm activity of the extracted fungal pigments for the tested bacterial, and fungal isolates as colorimetrically quantified at wavelength 570 nm by crystal violet stain

*T. purpurogenus* was grown on PDB at 30 °C, for 10 days at static conditions, the cultures were extracted with different solvents “acetone, methylene chloride, methanol, ethanol, methyl ethyl ketone, chloroform, hexane, and ethylacetate. The activity of the extracts was tested against the multidrug resistant bacteria “*K. pneumonia, P. aeruginosa, E. coli, Enterobacter agglomerans, S. aureus*, *S. epidermidis* and *Bacillus subtilis*”, in addition to the pathogenic fungi “*Candida albicans* and *C. tropicalis”.* From the diameter of the inhibition zone (Table S1, Fig. 2), the highest activity was reported for *T. purpurogenus* ethyl acetate extract against *E. coli, K. pneumonia* (17.0 mm)*, P. aeruginosa, S. aureus* (16.2 mm), *S epidermidis* (14.2 mm), *E. agglomerans* (11.0 mm), in addition to their activity towards *C. albicans* (14 mm) and *C. tropicalis* (12 mm). Following to ethyl acetate extract, the methanolic and ethanolic extracts have a relatively higher activity towards the tested pathogenic microorganisms as revealed in Table S1. The minimum inhibitory concentration (MIC) of the *T. purpurogenus* ethyl acetate extracts was assessed from their different concentrations on the tested bacterial isolates in liquid media. From the MIC values (Table S2), the ethyl acetate extracts has the highest activity against *E. coli* (6.8 µg/ml), *S. aureus* and *C. albicans* (12.5 µg/ml), followed by mild activity against *K. pneumonia, P. aeruginosa, S. epidermidis*, and *C. tropicalis* (25.5 µg/ml)*,* with low activity against *E. agglomerans* (50.4 µg/ml) (Fig. 2B). Thus, the anti-biofilm activity of this ethyl acetate extract towards the susceptible microorganisms “*E. coli*, *S. aureus* and *C. albicans”* was assessed visually and colorimetrically at λ_600_ nm. The bacterial isolates were grown on LB broth media, and *C. albicans* was grown on Sabouraud dextrose broth media with the ethyl acetate extracts of *T. purpurogenus* (10.0 µg/ml), for 48 h, then the tubes were decanted, washed with distilled water, amended with crystal violet, parallel to the control media. The developed blue colored complex of the lipopolysaccharides matrix of the bacterial film, was visually observed, compared to the control cultures (Fig. 3). The anti-biofilm activity of the ethyl acetate extracts of *T. purpurogenus* was summarized in Table S3 and Fig. 3. The highest anti-biofilm activity of *T. purpurogenus* ethyl acetate extracts was recorded for *S. aureus* (86.2%), *C. albicans* (85.8%) and *E. coli* (79.7%), compared to the control (non-treated) cells. Thus, the higher biological activity of the ethyl acetate extracts *T. purpurogenus* against the tested multi-drug resistant bacteria could be mainly attributed to their biofilm activity.

### Chemical characterization of the extracted azaphilone pigments from *T. purpurogenus*

**Fig. 4:**
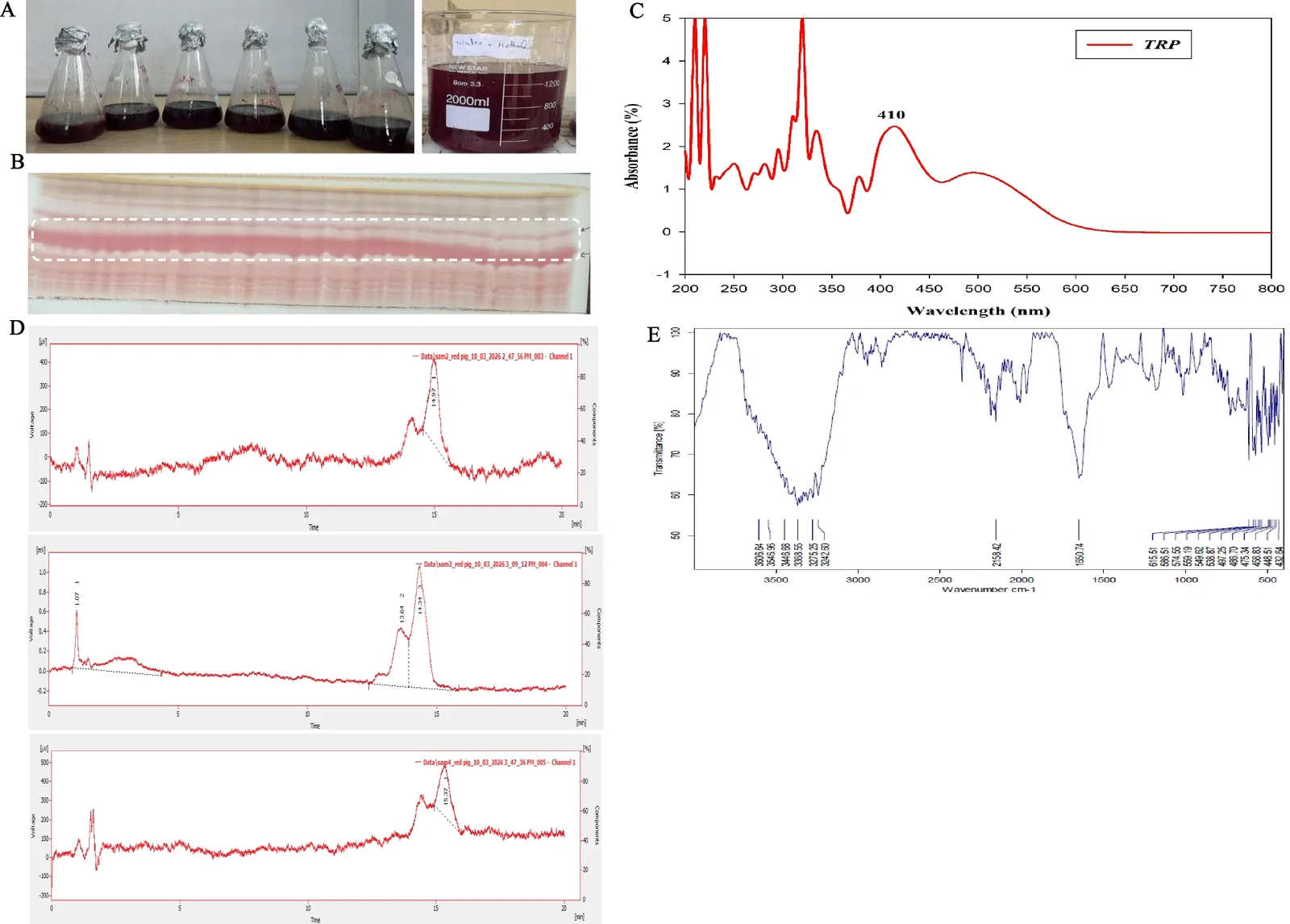
Extraction and chemical validation of the recovered pigments from *Talaromyces purpurogenus.* **A** Crude methanolic fungal extract. **B** TLC of the extracted pigments, prior to chemical validation. **C** HPLC chromatograms of the extracted and TLC-purified pigment (at different concentrations). **D** The UV-spectra of the TLC-purified pigments. **E** The FT-IR spectra of the purified pigment

The chemical identity of the ethyl acetate extract of *T. purpurogenus* grown on PDB broth (Cat. # DF0549-17–9) was resolved by the different chromatographic and spectroscopic analyses. The extract was fractionated by TLC (Fig. 4), the highly dense orange-red azaphilone pigment was recorded, scraped-off, and their chemical identity was further resolved by the UV–Vis, HPLC, HNMR and LC–MS/MS analyses to check their structural and chemical identities. The colorimetric properties of the purified sample was checked by UV–Vis analyses, the maximum absorbance was measured at λ_410_ nm (2.7 AU/ml), followed by slight shoulder at λ_510_ nm (1.5 AU/ml). The maximum absorbance at λ_410_ nm refers to the identity of the major compound as orange azaphilone pigment, compared to the red one (Fig. 4C). The chemical homogeneity of the eluted azaphilone pigment of *T. purpurogenus* was resolved from the HPLC chromatograms, at different pigment concentrations (1.0, 1.5 and 2.0 AUλ_410_/ml). From the HPLC chromatograms (Fig. 4D), the peak of orange azaphilone pigment was resolved at 14.9–15.5 min, with the exponential increase on peak area with the corresponding concentration ensuring the homogeneity of the TLC-purified pigment. From the analytical HPLC chromatogram, the purity of the target peak was 88% as calculated from the ratio of the main target peak to the total chromatographic peaks areas.

The functional groups of the compound were assessed by FT-IR analyses in the range of 500–4000 cm^−1^. A broad absorption band observed in the region of 3600–3240 cm^−1^ indicates the presence of hydroxyl (-OH) and/or amino (-NH) groups (Fig. 4). The absorption peak at 2164 cm^−1^ could be attributed to unsaturated functionalities or weak stretching vibrations of triple-bond-related groups. A strong and sharp absorption band at 1667 cm^−1^ corresponds to the carbonyl (C = O) stretching vibration, suggests the presence of ketone, or conjugated carbonyl moieties. Additionally, several peaks detected in the fingerprint region (1250–500 cm^−1^) are characteristic of C-O, C-N, and aromatic C-H bending vibrations, confirming the complex aromatic and oxygenated nature of the compound. Overall, the FT-IR data support the presence of hydroxylated and carbonyl-containing bioactive secondary metabolites.

**Fig. 5:**
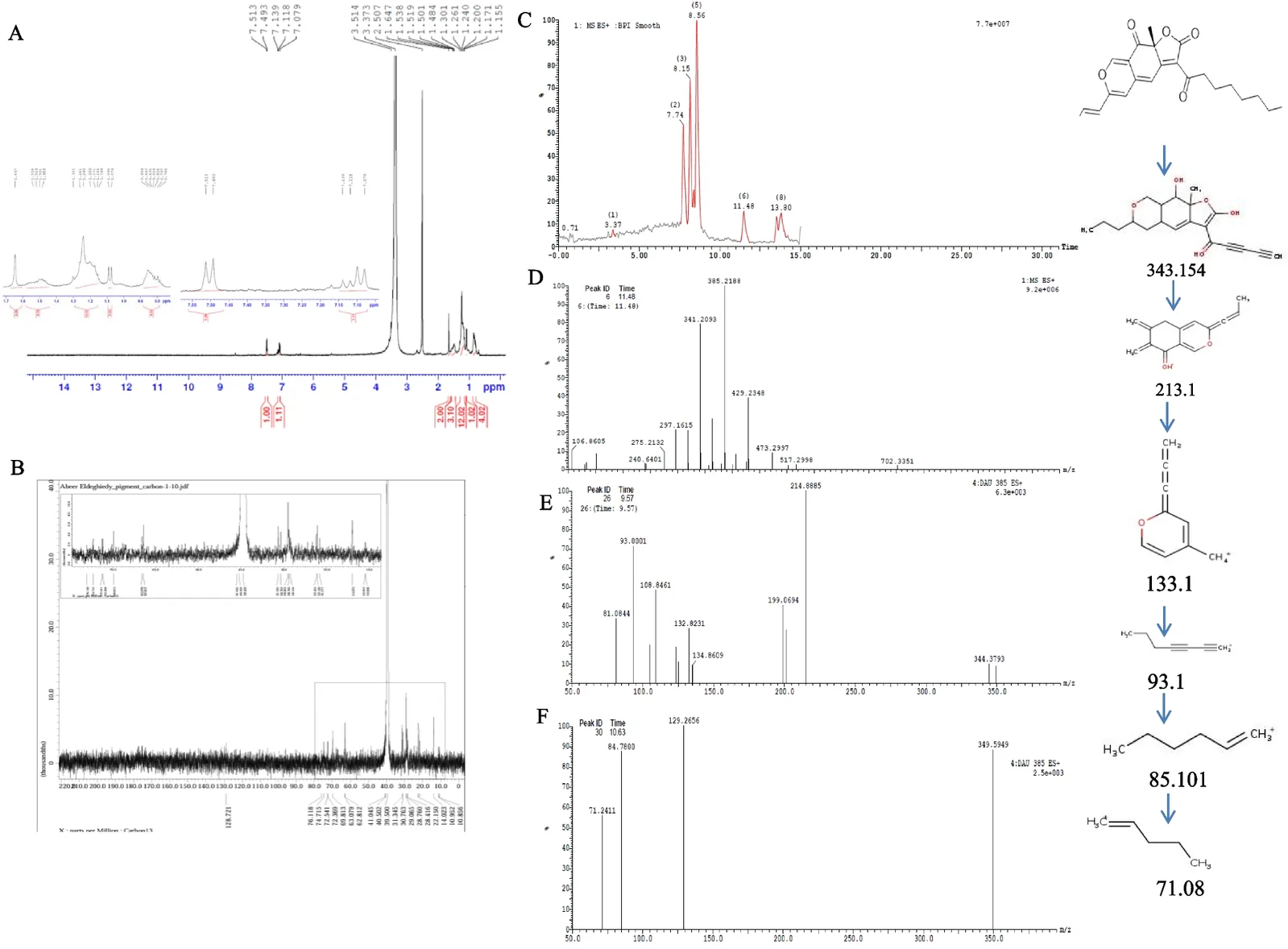
Spectroscopic validation of the purified orange pigment by H-NMR and LC–MS/MS. The H-NMR spectra (**A**) and 13CNMR spectra (**B**) of the purified target compound. The LC–MS/MS spectra of the target compound as revealed from the Total Ion Chromatogram (TIC) (**C**), LC–MS (**D**), and MS–MS fragmentation pattern (**E**, **F**). The chemical structure of the sample was typically identical with dihydromonascorubrin, as an orange azaphilone pigment

The chemical identity of the purified compound was resolved from the HNMR spectroscopy. The HNMR spectra of the compound revealed characteristic proton signals support the presence of a conjugated aromatic polyketide structure related to dihydromonascorubrin or its derivatives (Fig. 5A). The signals observed in the aromatic/olefinic region at δ 7.08–7.51 ppm revealed the presence of conjugated aromatic or unsaturated protons, which are typical for azaphilone pigments containing extended π-electron systems. Strong signals appearing at δ 3.2–3.6 ppm corresponds to methoxy (-OCH₃) or oxygenated methylene protons adjacent to electronegative atoms. The multiple proton resonances in aliphatic region between δ 1.1–2.4 ppm are attributable to methyl and methylene groups associated with the side chains of the pigment skeleton. The absence of highly downfield aldehydic proton signals (> 9 ppm) suggests the predominance of conjugated ketone/ester functionalities rather than free aldehyde groups. Overall, the HNMR spectrum supports the presence of an oxygenated, highly conjugated azaphilone-type secondary metabolite, consistent to dihydromonascorubrin -like fungal pigments.

The 13CNMR spectrum of the isolated metabolite displayed multiple resonances distributed over the range of approximately δC 10–220 ppm, indicating the presence of structurally diverse carbon environments (Fig. 5B). Signals observed in the aliphatic region (δC ~ 10–50 ppm) may be attributed to methyl and methylene carbons, whereas resonances in the oxygenated/aliphatic and unsaturated carbon regions (δC ~ 50–100 and 100–160 ppm, respectively) correspond to the carbons associated with the oxygenated and conjugated framework of an azaphilone-type metabolite. Overall, the spectral profile refers to a highly substituted polyketide-type structure and support the tentative assignment of *Monascus* azaphilone pigment family, including dihydromonascorubrin. Thus, the compound is proposed as dihydromonascorubrin based on the combined chromatographic and spectroscopic evidences.

The identity of purified compound was verified by the LC–MS/MS. The MS/MS spectra of the target compound were revealed from the total ion chromatogram (Fig. 5C). From the 1 st MS spectra, the molecular mass of the compound was resolved at 385.2 m*/z* that being matched to the molecular mass dihydromonascorubrin azaphilone pigment (Fig. 5). Furthermore, the molecular fragmentation pattern of the selected peaks of the parent molecule (385.2 m*/z*), were analyzed by ESI–MS/MS. The molecular fragments of the parent molecule were of 343.1, 213.1, 133.1, 93.1, 85.1 and 71.08 m/z, that was being matched to the in silico fragmentation pattern of dihydro- monascorubrin by CFM-ID software. So, the purified compound of 385.2 m/z was matched to the molecular mass of reduced monascorubrin (dihydromonascorubrin) compared 382.45 m*/z* of authentic dihydromonascorubrin.

### Bioprocessing of pigment production by *T. purpurogenus* with Plackett–Burman design

**Fig. 6:**
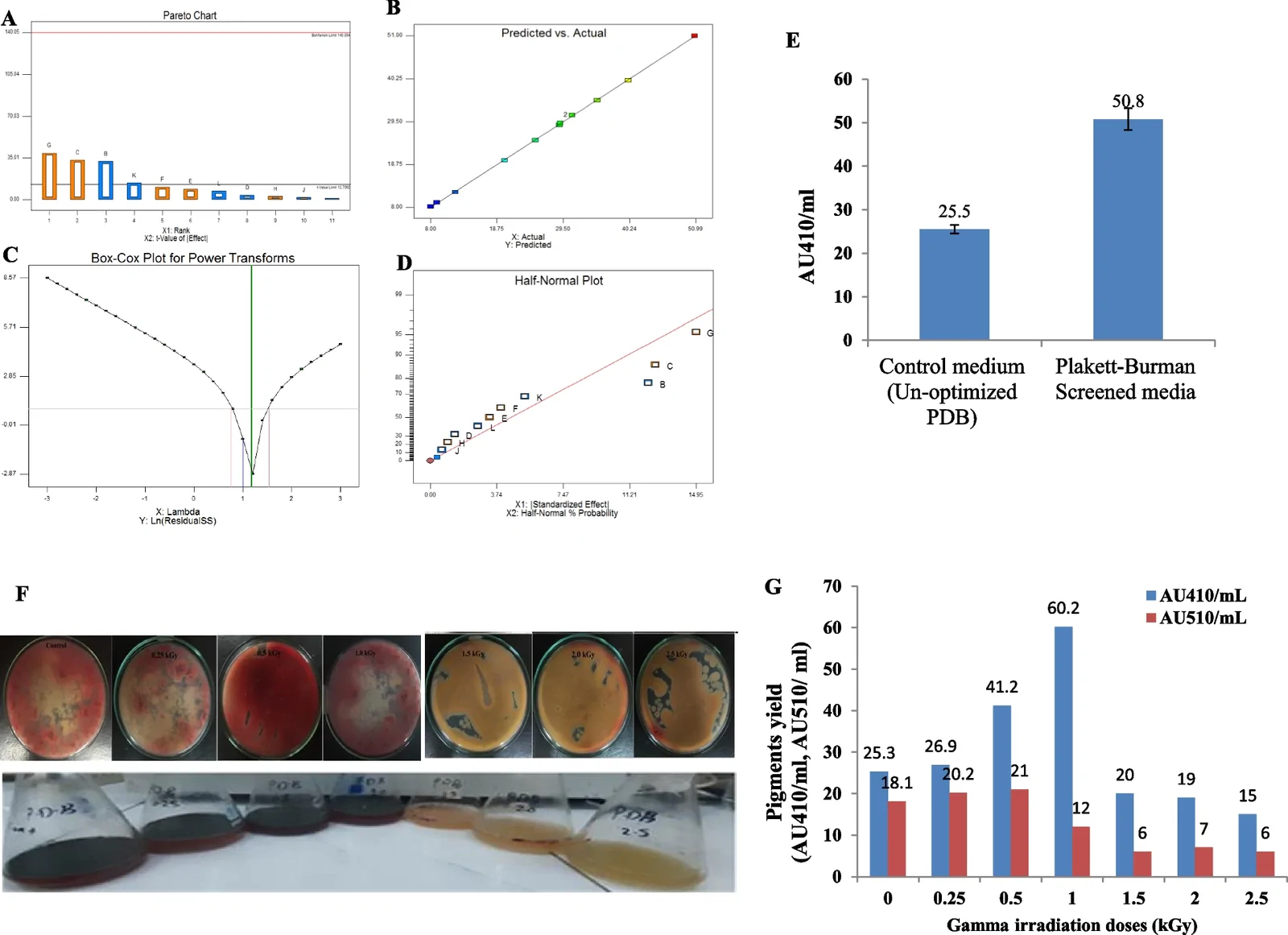
Nutritional optimization of *T. purpurogenus* culture medium for maximum pigment productivity by Plackett–Burman Design. The statistical parameters namely Pareto Chart (**A**), plots of predicted vs actual yield of pigment (**B**), plot of Box-Cox for power transform (**C**) and half-normal plot (**D**) for the orange pigment production by *T. purpurogenus*

The physicochemical parameters of *T. purpurogenus* were optimized to maximum their pigment productivity by Plackett–Burman design. Eleven independent variables were examined on the pigment productivity of *T. purpurogenus* (Table 2). The significance of variables affecting *T. purpurogenus* pigment productivity*,* as revealed from the predicted and actual values were summarized in the matrix of Plackett–Burman design (Table 3). The fluctuation of pigment productivity by *T. purpurogenus* as resolved from Plackett–Burman design was obviously wide ranged from 8.0 to 50.8 AU410/ml, reflecting the significance of parameters and efficiency of the design. The highest yield of azaphilone productivity (50.8 AU410/ml) by *T. purpurogenus* was recoded at the run number #12, with sucrose (8 g/l), glucose (4 g/l), fructose (8 g/l), yeast extract (4 g/l), malt extract (4 g/l), peptone (1 g/l), citric acid (0.5 g/l), sodium nitrate (2 g/l) at15 days incubation time, at static conditions. The lowest yield of pigment azaphilone (8.0 AU410/ml) by *T. purpurogenus* was reported at run # 1 that contains sucrose (8 g/l), glucose (4 g/l), fructose (4 g/l), yeast extract (1 g/l), malt extract (4 g/l), peptone (1 g/l), citric acid (2.0 g/l), sodium nitrate (5 g/l) of 15 days, at 150 rpm shaking incubation conditions. The correlation of pigment productivity and the independent variables concentrations was assessed by multiple-regression statistical analysis of variance (ANOVA) (Table 4). The variables regulating the pigment productivity by *T. purpurogenus* namely Pareto Chart, probability plot of the independent variables, plot of actual and predicted pigment yield was show in Fig. 6. The significance of each coefficient was determined from the *p*-value and student’s *t*-test. The "Pred R-Squared" of 0.9647 is in reasonable agreement with the "Adj R-Squared" of 0.9973 (Table 4). The “Adeq Precision" measures the signal to noise ratio, a ratio greater than 4 is desirable. The Adeq Precision ratio was 67.329 revealing the adequacy of the signal. From the half-normal probability plot, the points were arranged near the diagonal line, suggesting the perfect fitting of the pigment productivity response with the experimental variables. From the statistical analysis, the constructed model was highly significant as revealed from the values of Fisher’s *F*-test 407.9 and lower probability *p*-value 0.0385. The first order polynomial equation representing the pigment production by *T. purpurogenus* was represented by the following equation;

**Table 2:** Minimum and maximum ranges of the parameters of Plackett–Burman Design for nutritional optimization of the pigment production by *T. purpurogenus*

No	**Factors**	**Levels**	
		Minimum	Maximum
1	Incubation time (days)	7.0	15.0
2	Shaking speed (RPM)	0.0	120.0
3	pH	5.0	8.0
4	Sucrose (g/L)	4.0	8.0
5	Glucose (g/L)	4.0	8.0
6	Fructose (g/L)	4.0	8.0
7	Yeast Extract (g/L)	1.0	4.0
8	Malt Extract (g/L)	1.0	4.0
9	Peptone (g/L)	1.0	4.0
10	Citric acid (g/L)	0.5	2.0
11	Sodium nitrate (g/L)	2.0	5.0

**Table 3:** Matrix of the Plackett–Burman design with the predicted and actual values of pigment yield response at AU410/ml by *T. purpurogenus*

Run	**Incubation time (day)**	**Shaking speed rpm**	**pH**	**Sucrose (g/L)**	**Glucose (g/L)**	**Fructose (g/L)**	**Yeast Extract (g/L)**	**Malt Extract (g/L)**	**Peptone (g/L)**	**Citric acid (g/L)**	**Sodium nitrate (g/L)**	**Response AU410/ml**
1	7	120	8	8	4	4	1	4	1	2	5	8.5
2	15	120	8	4	4	4	4	1	4	2	2	9.6
3	15	1200	5	8	8	8	1	1	1	2	2	5.3
4	7	0	5	4	4	4	1	1	1	0.50	2	20.2
5	7	0	5	8	4	8	4	1	4	2	5	29.4
6	15	0	5	4	8	4	4	4	1	2	5	35.4
7	15	0	8	8	8	4	1	1	4	0.50	5	25.5
8	7	120	5	8	8	4	4	4	4	0.50	2	40.6
9	7	0	8	4	8	8	1	4	4	2	2	30.4
10	7	120	8	4	8	8	4	1	1	0.50	5	30.9
11	15	1200	5	4	4	8	1	4	4	0.50	5	28.9
12	15	0	8	8	4	8	4	4	1	0.5	2	50.8

**Table 4:** ANOVA for selected factorial model

Source	Sum of squares	df	Mean square	F-Value	*p*-value Prob > F	
Model	1798.342	10	179.8342	407.9414	0.0385	**Significant**
B-Shaking speed	450.1875	1	450.1875	1021.219	0.0199	
C-pH	480.0675	1	480.0675	1089	0.0193	
D-Sucrose	5.740833	1	5.740833	13.02268	0.1721	
E-Glucose	33.6675	1	33.6675	76.3724	0.0725	
F-Fructose	47.60083	1	47.60083	107.9792	0.0611	
G-Yeast Extract	670.5075	1	670.5075	1521	0.0163	
H-Malt Extract	2.900833	1	2.900833	6.58034	0.2366	
J-Peptone	1.2675	1	1.2675	2.875236	0.3392	
K-Citric acid	84.80083	1	84.80083	192.3648	0.0458	
L-Sodium nitrate	21.60083	1	21.60083	49	0.0903	
Residual	0.440833	1	0.440833			
Cor Total	1798.783	11				
Std. Dev	0.663953		R-Squared	0.999755		
Mean	26.625		Adj R-Squared	0.997304		
C.V. %	2.493719		Pred R-Squared	0.964709		
PRESS	63.48		Adeq Precision	67.32879		

The Model F-value of 407.94 implies the model is significant. There is only a 3.85% chance that a "Model F-Value" this large could occur due to noise. Values of "Prob > F" less than 0.0500 indicate model terms are significant. In this case B, C, G, K are significant model terms. Values greater than 0.1000 indicate the model terms are not significant. Std. Dev. 0.663953, R-Squared 0.999755, Mean 26.625, Adj R-Squared 0.997304, C.V. % 2.493719, Pred R-Squared 0.964709, PRESS 63.48, Adeq Precision 67.32879

$$\begin{aligned} &\mathrm{Yield}\;\mathrm{of}\;\mathrm{azaphilone}\;\mathrm{by}\;T\mathit.\mathit\;purpurogenus\;(\mathrm{AU}410/\mathrm{ml})\\ &=\;-8.75\;-0.102\;\times\;\mathrm{Shaking}\;\mathrm{speed}\\ &\quad +\;4.216\;\times\;\mathrm{pH}\;-0.345\;\times\;\mathrm{Sucrose}\\ &\quad +\;0.83\;\times\;\mathrm{Glucose}\;+\;0.99\;\times\;\mathrm{Fructose}\\ &\quad +\;4.98\;\times\;\mathrm{Yeast}\;\mathrm{Extract}\;+\;0.327\\ &\quad \times\;\mathrm{Malt}\;\mathrm{Extract}\;-0.216\;\times\;\mathrm{Peptone}\\ &\quad -3.54\;\times\;\mathrm{Citric}\;\mathrm{acid}\;-0.894\\ &\quad \times\;\mathrm{Sodium}\;\mathrm{nitrate} \end{aligned}$$

### Effect of γ- irradiation of *T. purpurogenus* on the azaphilone pigment productivity

The effect of γ-irradiation of *T. purpurogenus* on azaphilone productivity was assessed by irradiating their spores at different doses (0.25 to 2.5 kGy), then the pigments productivity was measured compared to the non-irradiated ones, at λ_410_ and λ_510_ nm. From the visual inspection of the plate culture of irradiated and non-irradiated spores (Fig. 6), an obvious increase to the pigment productivity upon irradiating the spores till 1.0 kGy, with a consequent decrease to the pigment productivity with the irradiating doses. The pigment productivity by *T. purpurogenus* was further quantified by growing on liquid media. A visual increase to the pigment productivity was observed by irradiating the spores at 1.0 kGy, followed by strong reduction to the pigment productivity with the higher irradiating doses. Practically, from the spectrophotometric analysis, the maximum absorption of the extracted pigment was measured at λ_410_ nm (60.2 AU410/ml), compared to λ_510_ nm (21.0 AU510/ml), for the fungal spores irradiated at 1.0 kGy (Fig. 6G), revealing the orange azaphilone pigment identity than red ones. Colormetrically, unlike the obvious increase to the orange pigment productivity with the spore γ-irradiation, the yield of red pigments “at λ510 nm” was relatively unaffected with the γ-irradiating doses.

### Antioxidant activity of the purified azaphilone pigment by *T. purpurogenus*

**Fig. 7:**
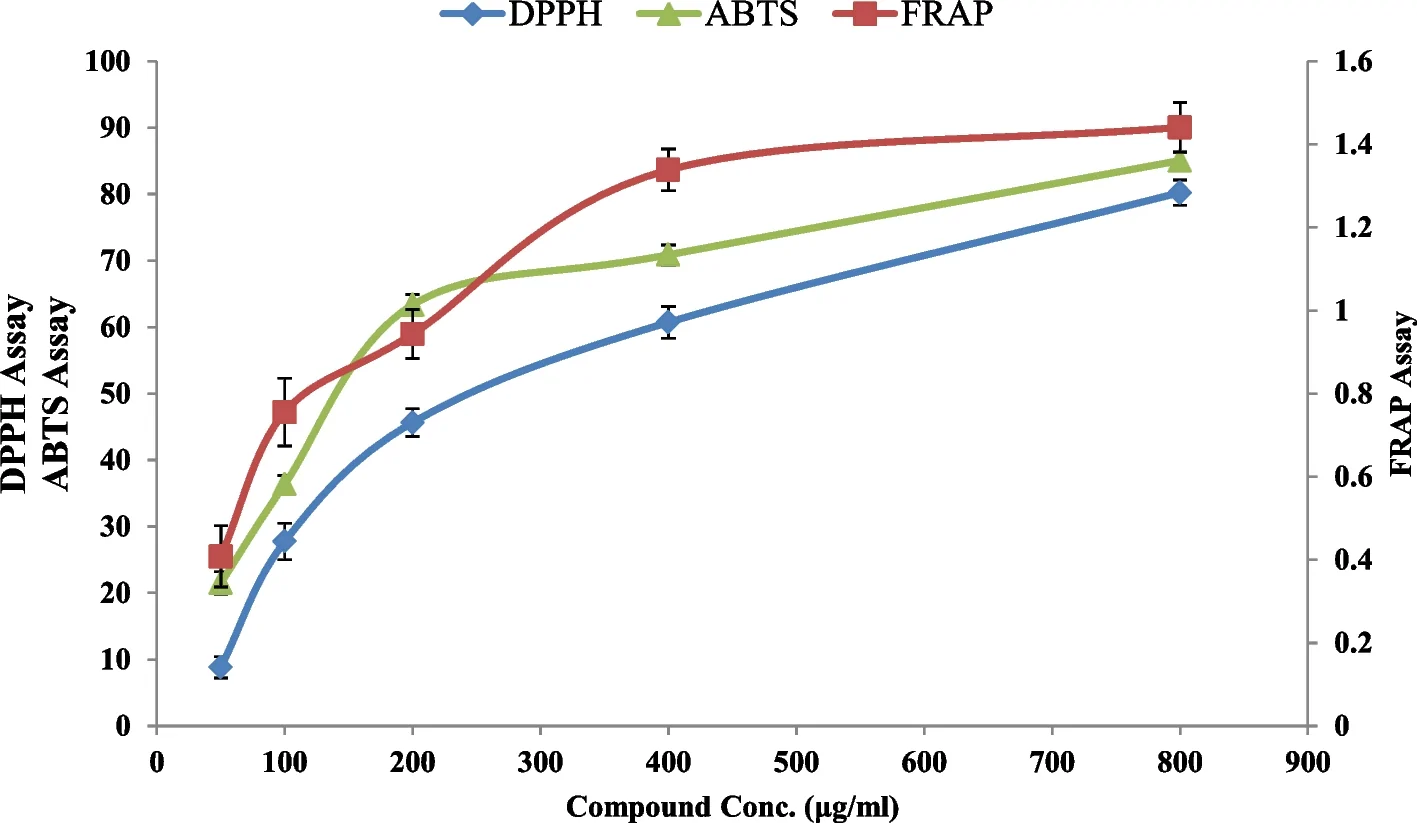
Antioxidant activity of the purified pigment of *T. purpurogenus* by DPPH, ABTS and FRAP assays

The antioxidant activity of the purified orange pigments of *T. purpurogenus* was increased in a concentration-dependent manner in all applied assays, revealing their potent free radical scavenging and reducing activities. The antioxidant activity of the orange pigments was increased progressively in a concentration-dependent manner by DPPH, FRAP and ABTS (Fig. 7). The higher antioxidant activity of the pigment by the ABTS and DPPH assays in dramatic concentration-dependent pattern ensures their strong free radical scavenging capacity. Also, the higher antioxidant activity of the pigment in a concentration-dependent pattern by the FRAP assay, ensures their powerful reducing efficiencies by single electron transfer (SET) reaction. The different assays suggest the remarkable antioxidant potential of the orange pigments as free radical scavenging and reducing activities.

### Pharmmapper-derived microbial target space of dihydromonascorubrin

PharmMapper-based target fishing was performed to generate the preliminary protein-target space of dihydromonascorubrin. This filtration yielded 55 microbial PharmMapper-derived hits, classified into 15 direct *E. coli* hits, 33 related bacterial hits, and 7 related fungal hits (Table S4).

The retained microbial target space was restricted to the experimented organisms, *E. coli*, *S. aureus*, and *C.albicans*. Only UniProtKB/Swiss-Prot entries showing exact organism-specific target matches or direct components of the corresponding enzyme complex were retained. This strict filtration produced final organism-specific candidate target sets comprising 80 targets for *E. coli*, 24 targets for *S. aureus*, and 11 targets for *C. albicans* (Tables S5-S7). These final candidate microbial targets were used for subsequent organism-specific network construction and docking target prioritization.

### Organism-specific PPI network construction and hub target prioritization

**Fig. 8:**
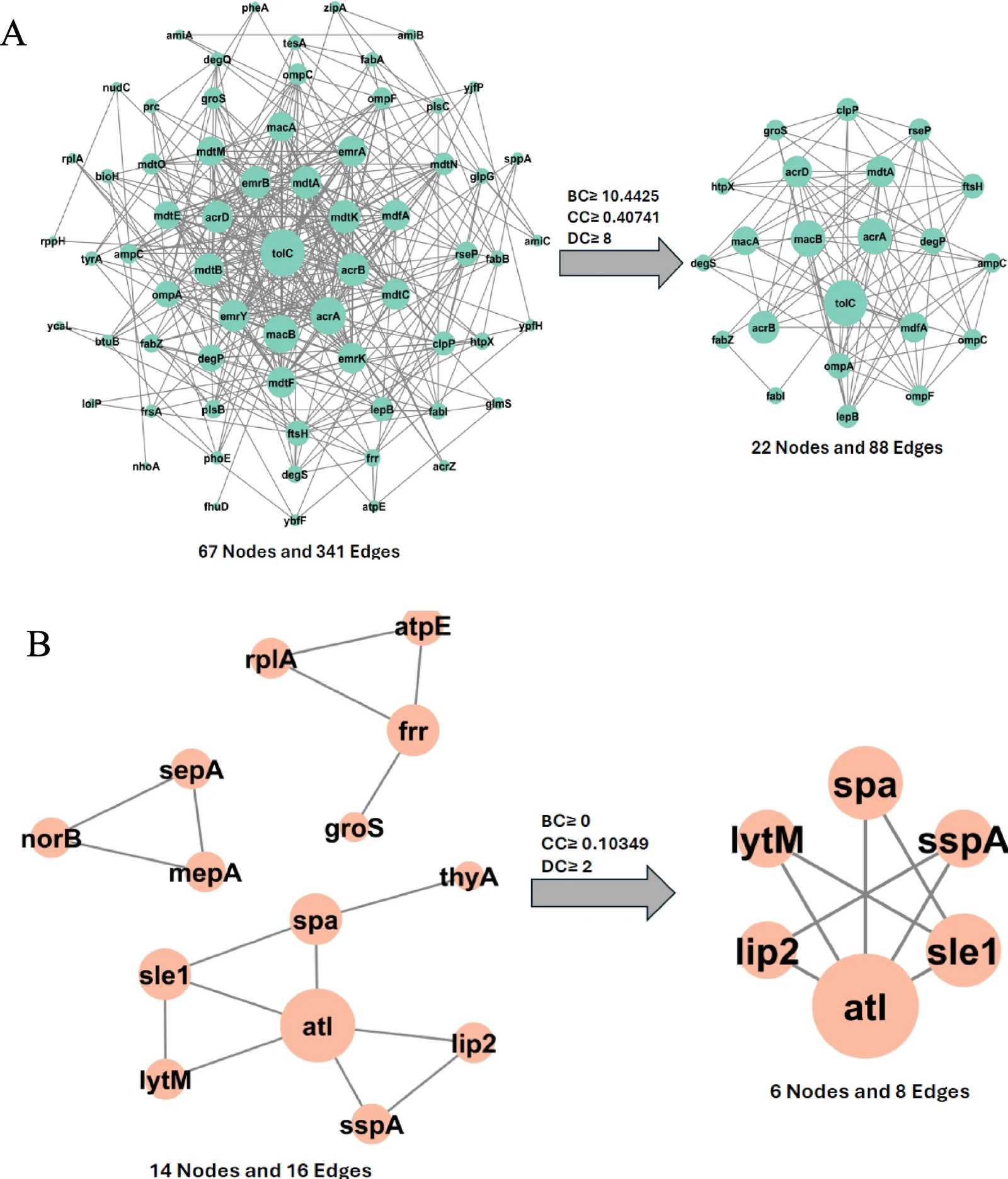
PPI network and hub-target filtration of Dihydromonascorubrin-predicted *E. coli* and *S. aureus* targets. **A** STRING analysis of the Dihydromonascorubrin-predicted of *E. coli* candidate targets generated a PPI network of 67 nodes and 341 edges. CytoNCA median-based filtration using BC ≥ 10.4425, CC ≥ 0.40741, and DC ≥ 8 retained a hub network of 22 nodes and 88 edges. Node size reflects degree centrality. **B** STRING analysis of the Dihydromonascorubrin-predicted *S. aureus* candidate targets generated a connected component of 14 nodes and 16 edges. CytoNCA median-based filtration using BC ≥ 0, CC ≥ 0.10349, and DC ≥ 2 retained a hub network of 6 nodes and 8 edges. Node size reflects degree centrality

The final organism-specific candidate target sets were submitted to STRING for PPI network construction using the corresponding microbial organism as background. For *E. coli*, the candidate target set generated a connected interaction network suitable for topological analysis, comprising 67 connected nodes and 341 edges after hiding disconnected nodes for visualization (Fig. 8). The interaction data were then imported into Cytoscape, and centrality parameters were calculated using CytoNCA. Median-based filtration using BC, CC, and DC retained 22 hub targets in the *E. coli* network (Fig. 8). The retained targets were arranged according to descending DC values to highlight their relative connectivity within the network (Table 5). TOLC emerged as the top-ranked *E. coli* hub, followed by highly connected efflux-associated targets including ACRA, MACB, and ACRB (Table 6). The dominant module was related to multidrug efflux and resistance-associated transport, represented by ACRA, ACRB, ACRD, TOLC, MACA, MACB, MDFA, and MDTA, which collectively form or contribute to efflux systems involved in the extrusion of toxic compounds and antibiotics, thereby supporting intrinsic and acquired multidrug resistance [74, 75]. A second module involved outer membrane permeability and envelope integrity, including OMPF, OMPC, and OMPA [76, 77]. Additional hubs were associated with envelope stress and protease systems, represented by LEPB, DEGS, RSEP, DEGP, CLPP, FTSH, and HTPX [78–82], whereas FABI and FABZ represented the fatty-acid biosynthesis module [83]. AMPC and GROES were retained as resistance- and protein-folding-related hubs, respectively. Collectively, the *E. coli* hub profile suggests that dihydromonascorubrin may affect efflux-mediated resistance, membrane permeability, envelope homeostasis, and lipid biosynthesis.

**Table 5:** Receptor source, docking-site definition, and grid parameters used for monascorubrin docking

Organism	**Target**	**Structure/model ID**	**Grid center (x, y, z)**	**Box dimensions (Å)**
*E. coli*	TolC	PDB: 1EK9	32.099, 84.249, 62.881	35 × 35 × 35
*E. coli*	AcrB	PDB: 4DX5	35.750, − 60.849, − 41.807	30 × 30 × 30
*S. aureus*	ATL/AmiA	PDB: 4KNL	7.868, − 21.703, 76.535	30 × 30 × 30
*S. aureus*	LYTM	PDB: 4ZYB	7.236, 57.257, 69.740	20 × 20 × 20
*C. albicans*	LIP4	AF-Q9P8W1-F1	4.184, − 1.8388, − 0.855646	22 × 26 × 28
*C. albicans*	LIP5	AF-Q9P8W0-F1	4.91149, − 2.238, − 1.46319	29 × 26 × 26

**Table 6:** Core bacterial targets prioritized by topological analysis. BC, CC, and DC represent betweenness centrality, closeness centrality, and degree centrality, respectively

Organism	**Target (Full Name)**	**Gene Symbol**	**BC**	**CC**	**DC**
*E. coli*	Outer membrane protein TolC	TOLC	1220.208	0.554622	30
	Multidrug efflux pump subunit AcrA	ACRA	160.7267	0.478261	23
	Macrolide export ATP-binding/permease protein MacB	MACB	396.5216	0.492537	23
	Multidrug efflux pump subunit AcrB	ACRB	18.02768	0.428571	20
	Multidrug resistance protein MdtA	MDTA	223.7256	0.478261	19
	Aminoglycoside efflux pump AcrD	ACRD	18.09482	0.431373	19
	Multidrug transporter MdfA	MDFA	109.4565	0.471429	18
	Macrolide export protein MacA	MACA	10.44246	0.420382	17
	Periplasmic serine endoprotease DegP	DEGP	450.7836	0.52381	15
	Outer membrane protein A	OMPA	342.2292	0.503817	15
	ATP-dependent zinc metalloprotease FtsH	FTSH	285.1397	0.437086	14
	Signal peptidase I/Leader peptidase I	LEPB	507.8312	0.478261	13
	Regulator of sigma-E protease RseP	RSEP	295.8796	0.458333	13
	ATP-dependent Clp protease proteolytic subunit	CLPP	144.3243	0.431373	13
	Outer membrane porin F	OMPF	72.99273	0.452055	11
	Outer membrane porin C	OMPC	453.5194	0.485294	11
	Co-chaperonin GroES	GROS	99.55848	0.415094	11
	Beta-lactamase/Cephalosporinase AmpC	AMPC	26.53868	0.431373	10
	3-hydroxyacyl-[acyl-carrier-protein] dehydratase FabZ	FABZ	162.0675	0.407407	9
	Serine endoprotease DegS	DEGS	57.47035	0.4125	8
	Enoyl-[acyl-carrier-protein] reductase FabI	FABI	471.7663	0.455172	8
	Protease HtpX	HTPX	104.9678	0.428571	8
*S. aureus*	Bifunctional autolysin Atl	ATL	18	0.12381	5
	Staphylococcal protein A/Immunoglobulin G-binding protein A	SPA	10	0.121495	3
	N-acetylmuramoyl-L-alanine amidase Sle1	SLE1	2	0.121495	3
	Glutamyl endopeptidase/Staphylococcal serine proteinase V8	SSPA	0	0.119266	2
	Glycyl-glycine endopeptidase LytM	LYTM	0	0.119266	2
	Lipase 2/Glycerol ester hydrolase 2	LIP2	0	0.119266	2

For *S. aureus*, 14 of the 22 final candidate targets formed a connected STRING component, comprising 14 nodes and 16 edges (Fig. 8). CytoNCA-based topological analysis of this component retained six hub targets, these targets were arranged according to descending DC values, with ATL emerging as the top-ranked *S. aureus* hub, followed by SPA and SLE1. SSPA, LYTM, and LIP2 were retained as additional core targets (Table 6). Functionally, these hubs were grouped into four main categories: surface adhesion and immune evasion represented by SPA [84], secreted protease-mediated virulence modulation represented by SSPA [85], cell-wall remodeling and autolysin activity represented by ATL, SLE1, and LYTM [84, 86, 87], and lipase-related virulence functions represented by LIP2 [88, 89]. This hub profile suggests that dihydromonascorubrin may interfere with virulence-associated surface proteins, extracellular proteolysis, cell-wall remodeling, and lipid-associated functions in *S. aureus*. In contrast, the *C. albicans* candidate targets showed limited STRING connectivity, with only LIP4 and LIP5 forming a connected interaction pair. Accordingly, LIP4 and LIP5 were prioritized as focused *Candida* docking targets, as they were the only network-supported proteins within the reviewed *C. albicans* candidate target set.

### Molecular docking analysis of dihydromonascorubrin against prioritized microbial targets

**Fig. 9:**
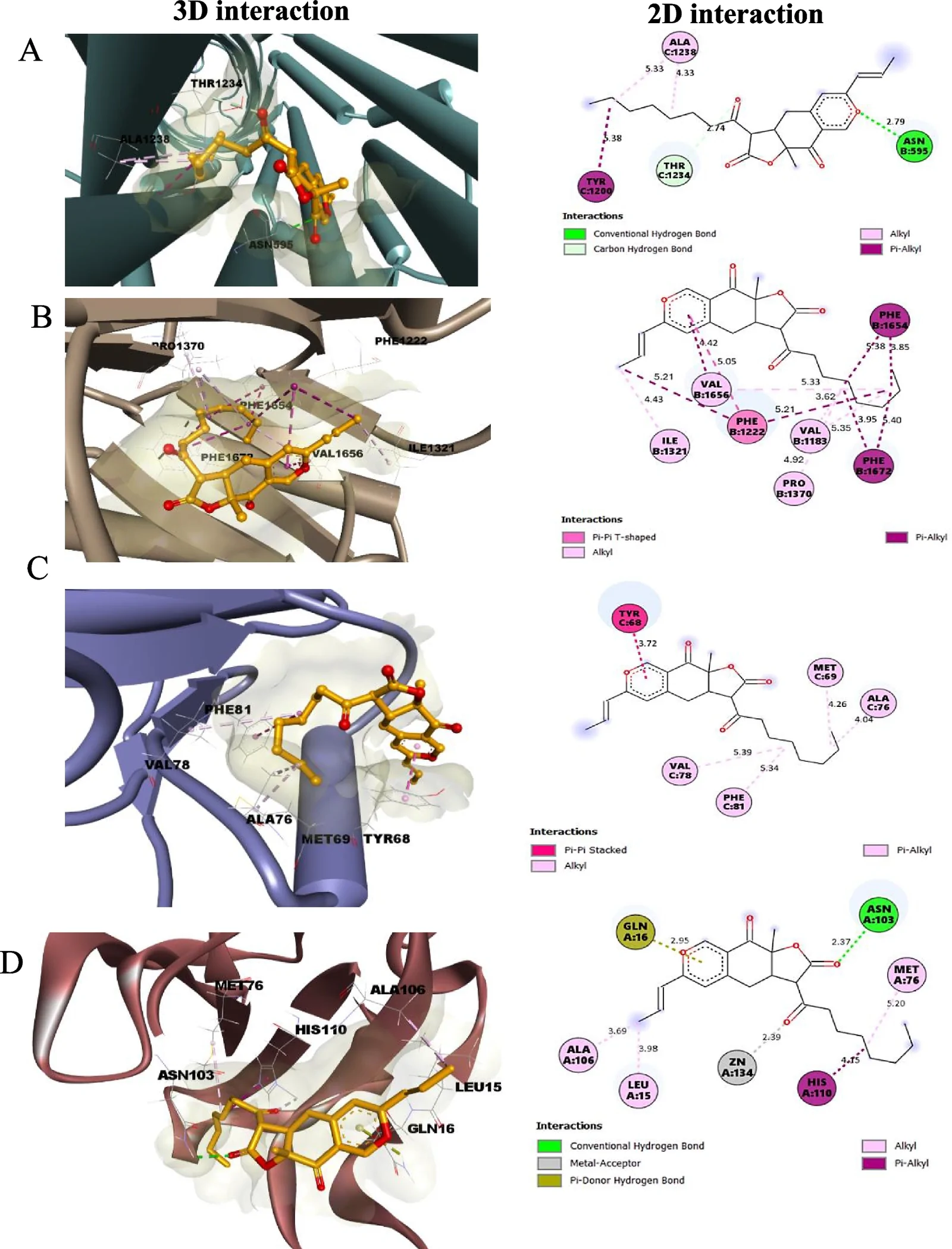
2D, and 3D docking interaction profiles of dihydromonascorubrin with the prioritized microbial targets. For each target, the left subpanel shows the 3D binding pose within the predefined docking region, and the right subpanel shows the corresponding 2D interaction map. The interaction of dihydromonascorubrin with *E. coli* TolC (**A**), AcrB (**B**), and *S. aureus* ATL/AmiA (**C**), and LYTM (**D**)

Dihydromonascorubrin showed favorable binding affinities across the prioritized bacterial and fungal docking targets, with predicted scores ranging from −7.0 to −9.6 kcal/mol (Table 7). Redocking of the corresponding co-crystallized ligands/substrate analogues successfully reproduced near-native crystallographic poses for AcrB, AtlA/AmiA, and LytM, with minimum RMSD values of 0.932, 1.699, and 1.765 Å, respectively, all within the ≤ 2.0 Å acceptance criterion. The bacterial docking results supported potential interactions with efflux-associated targets in *E. coli* and cell-wall remodeling targets in *S. aureus*, whereas the fungal docking results provided supportive exploratory evidence for interaction with the predicted catalytic regions of *C. albicans* lipases. The combined 3D binding poses and 2D interaction maps are presented in Fig. 9. For *E. coli*, dihydromonascorubrin showed moderate binding to TolC (−7.0 kcal/mol) and stronger binding to AcrB (−9.6 kcal/mol). In TolC, the ligand was positioned near the Asp371/Asp374-guided periplasmic entrance region and formed carbon hydrogen-bond contacts with THR A:366, together with an alkyl interaction with VAL C:1226. In AcrB, the compound was accommodated within the multidrug-binding pocket and was stabilized by multiple hydrophobic and aromatic contacts involving VAL B:1183, PHE B:1222, ILE B:1321, ALA B:1323, PRO B:1370, PHE B:1654, VAL B:1656, PHE B:1659, and PHE B:1672. For *S. aureus*, dihydromonascorubrin showed favorable binding to ATL/AmiA and LYTM, with docking scores of −7.0 and −7.4 kcal/mol, respectively. In ATL/AmiA, the ligand was stabilized within the peptidoglycan-binding groove through *π*–*π* interaction with TYR C:68 and hydrophobic contacts with MET C:69 and ALA C:76. In LYTM, the ligand was positioned within the catalytic groove and interacted with functionally relevant active-site components, including GLN A:16, Zn A:134, and ASP A:31, in addition to aromatic and hydrophobic contacts with LEU A:15, TYR A:21, TYR A:28, MET A:76, HIS A:108, and HIS A:110.

**Table 7:** Docking scores and interaction profiles of monascorubrin with prioritized microbial targets

Organism	**Target**	**Docking Score (kcal/mol)**	**Interaction Type**	**Interacting Residues (Amino Acid)**	**Bond Distance (Å)**
*E. coli*	TolC	− 7.0	Carbon H-bond	THR A:366	2.51 and 3.65
			Alkyl	VAL C:1226	5.36
*E. coli*	AcrB	− 9.6	Alkyl	VAL B:1183	3.75 and 5.43
				ILE B:1321	4.97
				ALA B:1323	3.99
				PRO B:1370	4.89
				VAL B:1656	4.64 and 5.23
			*π*-Alkyl	PHE B:1222	4.97 and 5.36
				PHE B:1654	3.82 and 5.34
				VAL B:1656	4.55
				PHE B:1672	3.83 and 5.49
			*π*-*π* Stacked	PHE B:1222	5.06
				PHE B:1659	4.70
*S. aureus*	ATL/AmiA	− 7.0	Alkyl	MET C:69	4.52
				ALA C:76	3.59 and 4.65
			*π*-Alkyl	TYR C:68	4.86 and 4.91
			*π*-*π* Stacked	TYR C:68	4.00
*S. aureus*	LYTM	− 7.4	Conventional H-bond	GLN A:16	2.10
			Metal-acceptor	Zn A:134	2.65
			*π*-Anion	ASP A:31	4.26
			*π*-*π* Stacked	TYR A:21	5.90
			*π*-*π* T-shaped	HIS A:108	5.94
			Alkyl	LEU A:15	3.65
				MET A:76	4.96
			*π*-Alkyl	TYR A:28	4.91
				HIS A:110	4.01
*C. albicans*	LIP4	− 8.4	Conventional H-bond	SER A:194	2.80
			Carbon hydrogen bond	HIS A:376	2.79
			Alkyl	VAL A:129	3.64
				ILE A:377	4.92
				VAL A:459	5.22
			*π*-Alkyl	PHE A:56	5.14
				PHE A:102	4.85
				ILE A:455	4.92
*C. albicans*	LIP5	− 9.2	Alkyl	LEU A:282	4.63
				ILE A:283	3.83
				VAL A:286	4.19
				LEU A:377	4.75
				VAL A:447	4.03
				ILE A:452	4.20
				LEU A:455	4.19 and 5.06
			*π*-Alkyl	PHE A:125	4.67
				VAL A:126	4.11
				ALA A:129	5.12
				PHE A:242	4.84 and 5.26

For *C. albicans*, docking using AlphaFold-predicted LIP4 and LIP5 models yielded favorable binding affinities of −8.4 and −9.2 kcal/mol, respectively. In LIP4, the ligand interacted with SER A:194 and HIS A:376, supporting ligand placement near the predicted catalytic lipase region defined by the conserved GYSGG motif. In LIP5, despite the stronger docking score, the interaction profile was dominated by hydrophobic contacts with PHE A:125, VAL A:126, ALA A:129, PHE A:242, LEU A:282, ILE A:283, VAL A:286, LEU A:377, VAL A:447, ILE A:452, and LEU A:455. Therefore, the LIP5 result was interpreted cautiously as evidence of favorable accommodation within the predicted lipase pocket rather than definitive catalytic-site inhibition.

## Discussion

The antimicrobial resistance of microorganisms usually attributed for the human irresponsible overuses and misuses of antibiotics in clinical treatment, agricultural practices and animal healthcare manifest their clinical consequences with the different human diseases [6]. Additionally, the patients infected with multi-drug resistant bacteria are more vulnerable to severe consequences than patients infected with non-MDR organisms [90]. Recently, the fungal pigments were emerged as one of the prominent natural compounds with potential biological activities towards the multi-drug resistance pathogenic microorganisms [91]. So, the objective of this study was to isolate a robust pigment-producing fugal isolate, maximize their pigment productivity, evaluating their biological activities and their mode of actions by the in silico analyses. Among the endophytes of *E. guineensis, Talaromyces purpurogenus* EFBL-2026, PZ292970 had the highest pigment-producing potency as 25.5 and 18.2 AU410/ml, and AU510, respectively. The highest absorption at the wavelengths 410 nm and 510 nm, integrated with the visual color, ensuring the presence orange-yellow, and red-pigments on the fungal extracts [45, 64, 65, 92]. *Talaromyces* species have been reported as well-known natural pigments producers, that closely similar to *Monascus* species [93, 94]. Chemically, *Talaromyces*/*Monascus* pigments encompassing a class of compounds of diverse compounds such as azaphilones, a fungal polyketide secondary metabolites, with a bicyclic pyranoquinone backbone [95]. Six categories of the pigments were produced by *Monascus* species namely the yellow pigments “monascin and ankaflavin”, orange pigments “rubropunctatin and monascorubrin”, and the red pigments “rubropunctamine and monascorubramine” [96, 97]. *Talaromyces* species were reported to secrete diverse amounts of *Monascus* like pigments, with undetectable mycotoxins [45, 72]. However, the chemical structural identity of *Talaromyces* species pigments was partially characterized compared to *Monoascus* species.

To fractionally precipitate *T. purpurogenus* pigments*,* different organic extracts was tested, and their activity against the multidrug resistant bacteria “*K. pneumonia, P. aeruginosa, E. coli, Enterobacter agglomerans, S. aureus*, *S. epidermidis* and *B. subtilis*”, and fungi “*C. albicans* and *C. tropicalis”,* were assessed. The highest activity was reported for *T. purpurogenus* ethyl acetate extract against all the tested MDR bacterial and fungal isolates, followed by methanolic and ethanolic extracts, as revealed from their MIC values. Similar extraction system for pigments of *T. purpurogenus* was used (Thiyam et al., 2020). To confirm antimicrobial efficiency, the highest antibiofilm activity of *T. purpurogenus* ethylacetate extract was assessed for *S. aureus* (86.2%), *C. albicans* (85.8%) and *E. coli* (79.7%), compared to the control. The antimicrobial activity of the *T. purpurogenus* pigments was coincident with those reported for different Gram-negative and Gram-positive bacteria [98–100]. The hydrophilicity of the extracted pigments of *T. purpurogenus* gave these compounds superiority for penetrating the bacterial cell walls of Gram-negative than Gram-positive ones and yeasts. In Gram-negative bacteria, the cell wall contains lipopolysaccharides (LPS), as a permeability barrier restricting the penetration of the hydrophobic compounds, ensuring the antimicrobial efficiency of this pigment, towards the MDR Gram-negative bacteria [98]. Coincidently, the red pigments of *Monascus* species have an effective antimicrobial activity [101]. The potential antifungal activity of *T. purpurogenus* extracts could be due to suppressing of conidial germination by disrupting the plasma membrane permeability, blocking of transport nutrients and respiration [101, 102]. Unlike to the higher efficiency of the extracted pigment of *T. purpurogenus* towards the Gran-negative bacteria, the pigment of *Monoascus* had a powerful activity against Gram-positive ones [103], assuming the unique structure–function relationships of *T. purpurogenus* pigments. Coincidently, *T. purpurogenus* orange pigment has a powerful activity towards *Staphylococcus aureus,* and *E. coli* [104–106].

The chemical identity of *T. purpurogenus* extract has been resolved by the different chromategra-phic and spectroscopic analyses. The purified sample has maximum absorbance at λ_410_ nm, with a slight shoulder at λ_510_ nm, assuming the major component an orange azaphilone pigment, compared to the red one. The purity of purified orange azaphilone pigment was checked by the HPLC at AUλ_410_/ml, gave a single peak at 15.0 min with a peak area in a concentration- dependent manner, ensuring the homogeneity of the purified pigment. The chemical identity of purified pigment was assessed by FT-IR analyses, gave a broad absorption band at 3600–3240 cm⁻^1^ for the hydroxyl and amino groups, the peak at 2164 cm^−1^ refers to weak stretching vibrations of triple-bond-related groups. From the FT-IR data and maximum absorption at λ_410_ nm, suggesting the compound as a conjugated azaphilone chromophore “dihydromonascorubrin pigments”. The red–orange coloration and visible-region absorption further strengthen this assignment. From the XRD pattern of purified compound, an intense diffraction peaks were observed at 2θ = 44°, 65°, and 78°, along with a few weaker reflections at lower angles, suggesting the presence of an ordered crystalline structure of high phase purity, with minimal amorphous content, supporting the identity of compound as dihydromonascorubrin or a closely related azaphilone derivative. The distinct diffraction peaks at approximately 2θ = 44°, 65°, and 78° indicates that the purified compound possesses crystalline domains, which is consistent with purified dihydromonascorubrin-like pigments [107]. Furthermore, the identity of the compound was resolved from HNMR spectroscopy, with a characteristic proton signals for the conjugated aromatic polyketide structure that related to dihydromonascorubrin. The signals in aromatic/olefinic region at δ 7.08–7.51 ppm ensure the conjugated aromatic/unsaturated protons, while, the multiple proton resonances in the aliphatic region between δ 1.1–2.4 ppm attributed to the methylene groups of the pigment skeleton side chains. Overall, from 1HNMR and 13CNMR spectra, the presence of an oxygenated, highly conjugated azaphilone-type secondary metabolite, substituted polyketide-type structure proposed the chemical identity as reduced monascorubrin “dihydromonascorubrin” [108].

The identity of the purified compound was verified by LC–MS, the target compound molecular mass was resolved with 385.2 m/z that matched to dihydromonascorubrin azaphilone pigment [109]. The molecular fragmentation pattern of the parent molecule (385.2 m/z), were analyzed by ESI–MS/MS, gave an identical molecular fragmentation pattern to dihydromonascorubrin, as verified from in silico fragmentation pattern of this compound by CFM-ID software. The highest yield of azaphilone by *T. purpurogenus* (50.8 AU410/ml) was recoded at the run number #12, with the sucrose (8 g/l), glucose (4 g/l), fructose (8 g/l), yeast extract (4 g/l), malt extract (4 g/l), peptone (1 g/l), citric acid (0.5 g/l), sodium nitrate (2 g/l) at 15 days incubation time, at static conditions. Overall, the yield of azaphilone pigment by *T. purpurogenus* was increased by about two folds upon nutritional optimization, compared to the non-optimized media. Similar studies were reported for the azaphilone pigment productivity by *Talaromyces* sp (Thiyam et al., 2020), [110]. The yield of pigments by γ-irradiated spores of *T. purpurogenus* was increased by ~ 1.2 folds, at absorption λ_410_ nm, at 1.0 kGy. The antioxidant activity of *T. purpurogenus* azaphilone pigment was obviously increased in a concentration-dependent manner for the DPPH, FRAP and ABTS, revealing their potential free radical scavenging and reducing activities. The different assays suggest the remarkable antioxidant potential of the orange pigments as free radical scavenging and reducing activities. Similarly, the antioxidant activities of the pigments from *Talaromyces purpurogenus* with free radical scavenging activity and reducing properties were reported [110, 111].

The in silico workflow provides a mechanistic framework for interpreting the antimicrobial and antibiofilm activities of dihydromonascorubrin. Organism-specific target prediction and PPI analysis prioritized the major microbial target modules, while molecular docking provided predictive structural support for selected network-supported targets by demonstrating favorable dihydromonascorubrin accommodation within functionally relevant binding or catalytic regions. In *E. coli*, the hub targets were concentrated around multidrug efflux systems, outer-membrane permeability proteins, envelope stress/protease components, and fatty-acid biosynthesis enzymes. TOLC emerged as the top-ranked hub, followed by several efflux-associated targets, including ACRA, MACB, and ACRB, that it was highly relevant to antibacterial susceptibility because Gram-negative bacteria depend strongly on outer-membrane integrity, porin-mediated permeability, and efflux-mediated extrusion of toxic compounds [74, 75, 112]. Docking was consistent with the potential relevance of this module, as monascorubrin was predicted to occupy a region near the Asp371/Asp374-guided periplasmic entrance of TolC and the AcrB multidrug-binding pocket. AcrAB–TolC efflux machinery could be an important network-supported mechanism contributing to the antibacterial action of dihydromonascorubrin. Efflux pumps and outer-membrane proteins are increasingly recognized as contributors not only to antimicrobial resistance, but also to bacterial adhesion, stress tolerance, extracellular matrix regulation, and biofilm maturation. Therefore, the identification of AcrAB–TolC-related targets, together with OMPF, OMPC, and OMPA, supports the possibility that dihydromonascorubrin may impair biofilm-associated tolerance by disturbing membrane transport and envelope homeostasis [77, 83, 84, 113, 114].

For *S. aureus*, the retained hubs were mainly associated with surface interaction, extracellular proteolysis, cell-wall remodeling, and lipid-related virulence functions. The prioritization of SPA suggests possible interference with surface-associated virulence and host interaction, while SSPA points to secreted protease-mediated pathogenicity. Moreover, ATL, SLE1, and LYTM are related to autolysin activity and peptidoglycan remodeling, processes that are important for cell separation, surface properties, and biofilm development in staphylococci. Docking predictions were consistent with this network interpretation, as dihydromonascorubrin was predicted to occupy the ATL/AmiA peptidoglycan-binding groove and the LYTM catalytic groove. The LYTM docking pose was particularly notable for interactions with the catalytic Zn^2^⁺ ion and neighboring active-site residues, supporting a potential effect on LytM-associated peptidoglycan processing. Thus, dihydromonascorubrin may affect *S. aureus* by mechanisms related to surface adhesion, cell-wall dynamics, and extracellular virulence-associated enzymes [84, 87, 88, 115, 116]. In *C. albicans*, the target set showed limited PPI connectivity, with LIP4 and LIP5 forming the only connected pair. Although this prevented hub-based topological ranking, the prioritization of these two lipase targets remains biologically meaningful because *Candida* lipases are associated with lipid utilization, tissue invasion, and fungal pathogenicity [117–120]. Docking against AlphaFold-predicted LIP4 and LIP5 models provided exploratory structural support for this lipase-related hypothesis. The LIP4 pose was more mechanistically informative because dihydromonascorubrin interacted with Ser194 and His376, supporting ligand placement near the predicted catalytic lipase region. Thus, the *Candida* findings provide exploratory support for a focused antifungal hypothesis potentially involving lipase-related functions. Overall, integration of target prediction, network analysis, and molecular docking suggests that dihydromonascorubrin may exert antimicrobial effects by organism-dependent, multi-target mechanisms. In *E. coli*, the dominant predicted mechanism is linked to efflux-associated resistance, membrane permeability, envelope homeostasis, and fatty-acid biosynthesis. In *S. aureus*, the results points to cell-wall remodeling, surface-associated virulence, extracellular proteolysis, and lipid-related virulence functions. In *C. albicans*, the findings suggest a more focused interaction with lipase-related pathogenicity factors.

In conclusion, *Talaromyces purpurogenus *# PZ292970.1, endophyte of *Elaeis guineensis*, has the highest productivity of dihydromonascorubrin derivatives, with a robust cytotoxic and antibiofilm activity towards the different multidrug-resistant bacteria “*E. coli, S. aureus, C. albicans”*. The chemical structure of the extracted pigment was resolved by TLC, HPLC, FT-IR, LC–MS/MS, NMR and XRD as a reduced monascorubrin-derivatives “dihydromonascorubrin” of 385.2 *m/z.* The yield of azaphilone pigment was increased by 2.5 folds upon nutritional bioprocessing by Plackett–Burman design, and γ-irradiation at 1.0 kGy. The purified dihydromonascorubrin pigment had a powerful total antioxidant activity with free radical scavenging and reducing properties. From the in silico analyses, integrating target prediction and molecular docking, dihydromonascorubrin exhibited multi-target mechanisms linked to the efflux-associated resistance, membrane permeability, fatty-acid biosynthesis in *E. coli*, and cell-wall remodeling, and surface-associated virulence in *S. aureus.* Thus, for the first time dihydromonascorubrin has been reported to be produced by *T. purpurogenus, *an endophyte of *E. guineensis,* with robust antimicrobial activities against the multidrug resistant bacteria, targeting the bacterial cell membrane efflux-associated proteins, envelope homeostasis and cell wall-remodeling enzymes in Gram-negative and Gram-positive MDR bacteria.

## Supplementary Information

Supplementary Material 1.

## Data Availability

The datasets generated and/or analyzed during the current study are included in the article/Supplementary Materials. The ITS sequence of the isolate Talaromyces purpurogenus has been deposited to the Genbank with accession # https://www.ncbi.nlm.nih.gov/nuccore/PZ292970.1
