# Supplementary material for: Bioprocessing of Talaromyces purpurogenus for production of dihydromonascorubrin with a powerful antioxidant and antimicrobial activities integrated with the network-guided target prediction and molecular docking analyses
Source: BMC Microbiol. 2026 Oct 22;26:909. doi: 10.1186/s12866-026-05707-z (PMC13688843; doi:10.1186/s12866-026-05707-z)
Supplement: Supplementary file 1 [file 12866_2026_5707_MOESM1_ESM.docx]

**Table S1. Antimicrobial activity of *T. purpurogenus* extracted pigment** **against different pathogenic bacterial:**

| **Pathogenic microbes** | | 1 | 2 | 3 | 4 | 5 | 6 | 7 | 8 | CAZ_30_/NS_100_ |
| --- | --- | --- | --- | --- | --- | --- | --- | --- | --- | --- |
| **Gram-negative bacteria** | *Klebsiella pneumonia* | -- | 10 | 17 | 9 | -- | 10 | -- | -- | 8 |
|  | *Pseudomonas aeruginosa* | -- | 9 | 16 | 8 | 8 | 8 | -- | -- | 9 |
|  | *Escherichia coli* | -- | -- | 17 | -- | -- | -- | -- | -- | 9 |
|  | *Enterobacter agglomerans* | -- | -- | 11 | 15 | -- | 16 | -- | -- | 17 |
| **Gram-positive bacteria** | *Staphylococcus aureus* | -- | -- | 16 | 14 | -- | 17 | -- | -- | 15 |
|  | *Staphylococcus epidermidis* | -- | -- | 14 | 13 | -- | 12 | -- | -- | -- |
|  | *Bacillus subtilis* | -- | -- | -- | 10 | -- | 9 | -- | -- | -- |
| **Unicellular fungi** | *Candida albicans* | 8 | -- | 14 | 15 | -- | 13 | -- | -- | -- |
|  | *Candida* *tropicalis* | 9 | -- | 12 | 14 | -- | 15 | -- | -- | -- |

The 10 days old cultures of *T. purpurogenus* was extracted with acetone (1), methylene chloride (2), ethylacetate (3), methanol (4), methyl ethyl ketone (5), ethanol (6), chloroform (7), hexane 8).

**Table S2. Minimum Inhibitory Concentration (MIC) Determination:**

|  | **Pathogenic microbes** | **(Red Pigment)** |
| --- | --- | --- |
| 1 | *Klebsiella pneumonia* | 25.5 |
| 2 | *Pseudomonas aeruginosa* | 25.5 |
| 3 | *Escherichia coli* | 6.8 |
| 4 | *Enterobacter agglomerans* | 50.5 |
| 5 | *Staphylococcus aureus* | 12.5 |
| 6 | *Staphylococcus epidermidis* | 25.8 |
| 7 | *Candida albicans* | 12.5 |
| 8 | *Candida tropicalis* | 25.6 |

1= 1000 ppm, 2= 500 ppm, 3= 250 ppm, 4= 125 ppm, 5= 62.5 and 6= 31.25 ppm

**Table S3.** The antibiofilm activity of *Talaromyces Red Pigment* by test tube methods

|  | Bacterial isolates | O.D of C.V stain @ 570.0nm Treat  (Control) | O.D Treat  (Red Pigment) | Inhibition % |
| --- | --- | --- | --- | --- |
| 1 | *Staphylococcus aureus* | 2.034 | 0.279 | 86.28% |
| 2 | *Candida albicans* | 0.789 | 0.112 | 85.81% |
| 3 | *Escherichia coli* | 1.071 | 0.217 | 79.74% |

**Table S4.** Classification of PharmMapper-derived microbial hits after UniProt-based organism annotation. Non-microbial entries were excluded, while bacterial and fungal hits were retained as the preliminary microbial target space.

| Target name | UniProt ID | Gene | Source organism | Microbial group |
| --- | --- | --- | --- | --- |
| Beta-lactamase (EC 3.5.2.6) (Cephalosporinase) (CSase) | P00811 | AMPC | *Escherichia coli* (strain K12) | Direct *E. coli* |
| Vitamin B12 transporter BtuB (Cobalamin receptor) (Outer membrane cobalamin translocator) | P06129 | BTUB | *Escherichia coli* (strain K12) | Direct *E. coli* |
| Cytidine deaminase (EC 3.5.4.5) (Cytidine aminohydrolase) (CDA) | P0ABF6 | CDD | *Escherichia coli* (strain K12) | Direct *E. coli* |
| Dr hemagglutinin structural subunit | P24093 | DRAA | *Escherichia coli* | Direct *E. coli* |
| 3-oxoacyl-[acyl-carrier-protein] synthase 1 (EC 2.3.1.41) (3-oxoacyl-[acyl-carrier-protein] synthase I) (Beta-ketoacyl-ACP synthase I) (KAS I) | P0A953 | FABB | *Escherichia coli* (strain K12) | Direct *E. coli* |
| Enoyl-[acyl-carrier-protein] reductase [NADH] FabI (ENR) (EC 1.3.1.9) (NADH-dependent enoyl-ACP reductase) | P0AEK4 | FABI | *Escherichia coli* (strain K12) | Direct *E. coli* |
| Iron(3+)-hydroxamate-binding protein FhuD (Ferric hydroxamate uptake protein D) (Ferrichrome-binding periplasmic protein) (Iron(III)-hydroxamate-binding protein FhuD) | P07822 | FHUD | *Escherichia coli* (strain K12) | Direct *E. coli* |
| Oxygen-insensitive NAD(P)H nitroreductase (EC 1.-.-.-) (Dihydropteridine reductase) (EC 1.5.1.34) (FMN-dependent nitroreductase) | P38489 | NFSB | *Escherichia coli* (strain K12) | Direct *E. coli* |
| NAD-capped RNA hydrolase NudC (DeNADding enzyme NudC) (EC 3.6.1.-) (NADH pyrophosphatase) (EC 3.6.1.22) | P32664 | NUDC | *Escherichia coli* (strain K12) | Direct *E. coli* |
| Outer membrane protein A (OmpA) (Outer membrane porin A) (Outer membrane protein 3A) (Outer membrane protein B) (Outer membrane protein II*) (Outer membrane protein d) | P0A910 | OMPA | *Escherichia coli* (strain K12) | Direct *E. coli* |
| Outer membrane porin F (Outer membrane protein 1A) (Outer membrane protein B) (Outer membrane protein F) (Outer membrane protein IA) (Porin OmpF) | P02931 | OMPF | *Escherichia coli* (strain K12) | Direct *E. coli* |
| Phospholipase A1 (EC 3.1.1.32) (EC 3.1.1.4) (Detergent-resistant phospholipase A) (DR-phospholipase A) (Outer membrane phospholipase A) (OM PLA) (OMPLA) (Phosphatidylcholine 1-acylhydrolase) | P0A921 | PA1 | *Escherichia coli* (strain K12) | Direct *E. coli* |
| Thioesterase 1/protease 1/lysophospholipase L1 (TAP) (Acyl-CoA thioesterase 1) (TESA) (EC 3.1.2.2) (Acyl-CoA thioesterase I) (Arylesterase) (EC 3.1.1.2) (Lysophospholipase L1) (EC 3.1.1.5) (Oleoyl-[acyl-carrier-protein] hydrolase) (EC 3.1.2.14) (Phospholipid degradation C) (Pldc) (Protease 1) (EC 3.4.21.-) (Protease I) (Thioesterase I/protease I) (TEP-I) | P0ADA1 | TESA | *Escherichia coli* (strain K12) | Direct *E. coli* |
| Thymidylate synthase (TS) (TSase) (EC 2.1.1.45) | P0A884 | TYSY | *Escherichia coli* (strain K12) | Direct *E. coli* |
| Cell division protein ZipA (FtsZ interacting protein A) | P77173 | ZIPA | *Escherichia coli* (strain K12) | Direct *E. coli* |
| Methylmalonyl-CoA carboxyltransferase 5S subunit (EC 2.1.3.1) (Transcarboxylase 5S subunit) | Q70AC7 | 5S | *Propionibacterium freudenreichii* subsp. *shermanii* | Related bacteria |
| Diacylglycerol acyltransferase/mycolyltransferase Ag85B (DGAT) (EC 2.3.1.122) (EC 2.3.1.20) (30 kDa extracellular protein) (Acyl-CoA:diacylglycerol acyltransferase) (Antigen 85 complex B) (85B) (Ag85B) (Extracellular alpha-antigen) (Fibronectin-binding protein B) (Fbps B) | P9WQP0 | A85B | *Mycobacterium tuberculosis* (strain CDC 1551 / Oshkosh) | Related bacteria |
| Diacylglycerol acyltransferase/mycolyltransferase Ag85B (DGAT) (EC 2.3.1.122) (EC 2.3.1.20) (30 kDa extracellular protein) (Acyl-CoA:diacylglycerol acyltransferase) (Antigen 85 complex B) (85B) (Ag85B) (Extracellular alpha-antigen) (Fibronectin-binding protein B) (Fbps B) | P9WQP1 | A85B | *Mycobacterium tuberculosis* (strain ATCC 25618 / H37Rv) | Related bacteria |
| Autolysin (EC 3.5.1.28) (Cell wall hydrolase) (Mucopeptide aminohydrolase) (Murein hydrolase) (N-acetylmuramoyl-L-alanine amidase) | P06653 | ALYS | *Streptococcus pneumoniae* serotype 4 (strain ATCC BAA-334 / TIGR4) | Related bacteria |
| Chorismate mutase AroH (EC 5.4.99.5) | Q84FH6 | AROH | *Thermus thermophilus* | Related bacteria |
| ATP synthase subunit c, sodium ion specific (ATP synthase F(0) sector subunit c) (F-type ATPase subunit c) (F-ATPase subunit c) (Lipid-binding protein) | P21905 | ATPL | *Propionigenium modestum* | Related bacteria |
| Beta-lactamase SHV-1 (EC 3.5.2.6) (PIT-2) (Sulfhydryl reagent variable 1) | P0AD64 | BLA1 | *Klebsiella pneumoniae* | Related bacteria |
| Catechol 1,2-dioxygenase (EC 1.13.11.1) (1,2-CTD) | P07773 | CATA | *Acinetobacter baylyi* (strain ATCC 33305 / BD413 / ADP1) | Related bacteria |
| Co-chaperonin GroES (10 kDa antigen) (10 kDa chaperonin) (BCG-A heat shock protein) (Chaperonin-10) (Cpn10) | P9WPE4 | CH10 | *Mycobacterium tuberculosis* (strain CDC 1551 / Oshkosh) | Related bacteria |
| Co-chaperonin GroES (10 kDa antigen) (10 kDa chaperonin) (BCG-A heat shock protein) (Chaperonin-10) (Cpn10) | P9WPE5 | CH10 | *Mycobacterium tuberculosis* (strain ATCC 25618 / H37Rv) | Related bacteria |
| Nicotinate-nucleotide--dimethylbenzimidazole phosphoribosyltransferase (NN:DBI PRT) (EC 2.4.2.21) (N(1)-alpha-phosphoribosyltransferase) | A9MTA4 | COBT | *Salmonella paratyphi* B (strain ATCC BAA-1250 / SPB7) | Related bacteria |
| Sterol 14alpha-demethylase (EC 1.14.15.36) (CYPLI) (Cytochrome P450 51) (Cytochrome P450-14DM) (Cytochrome P450-LIA1) (Sterol 14-alpha demethylase) | P9WPP9 | CP51 | *Mycobacterium tuberculosis* (strain ATCC 25618 / H37Rv) | Related bacteria |
| Camphor 5-monooxygenase (EC 1.14.15.1) (Cytochrome P450-cam) (Cytochrome P450cam) | P00183 | CPXA | *Pseudomonas putida* (*Arthrobacter siderocapsulatus*) | Related bacteria |
| 6-deoxyerythronolide B hydroxylase (6-DEB hydroxylase) (EC 1.14.15.35) (CYPCVIIA1) (Cytochrome P450 107A1) (Cytochrome P450eryF) (Erythromycin A biosynthesis hydroxylase) | Q00441 | CPXJ | *Saccharopolyspora erythraea* (strain ATCC 11635 / DSM 40517 / JCM 4748 / NBRC 13426 / NCIMB 8594 / NRRL 2338) | Related bacteria |
| DNA protection during starvation protein 2 (EC 1.16.-.-) | Q8RPQ2 | DPS2 | *Bacillus anthracis* | Related bacteria |
| Carboxylesterase 2 (EC 3.1.1.1) (Esterase II) | Q53547 | EST2 | *Pseudomonas fluorescens* | Related bacteria |
| 3-hydroxyacyl-[acyl-carrier-protein] dehydratase FabZ (EC 4.2.1.59) ((3R)-hydroxymyristoyl-[acyl-carrier-protein] dehydratase) ((3R)-hydroxymyristoyl-ACP dehydrase) (Beta-hydroxyacyl-ACP dehydratase) | O25928 | FABZ | *Helicobacter pylori* (strain ATCC 700392 / 26695) (Campylobacter pylori) | Related bacteria |
| Glutamate mutase sigma subunit (EC 5.4.99.1) (Glutamate mutase S chain) (Glutamate mutase small subunit) (Methylaspartate mutase) | Q05488 | GMSS | *Clostridium tetanomorphum* | Related bacteria |
| Lethal factor (LF) (EC 3.4.24.83) (Anthrax lethal toxin endopeptidase component) | P15917 | LEF | *Bacillus anthracis* | Related bacteria |
| Triacylglycerol lipase (EC 3.1.1.3) (Extracellular lipase) (Triacylglycerol ester hydrolase) | P22088 | LIP | *Burkholderia cepacia* (*Pseudomonas cepacia*) | Related bacteria |
| Neisserial autotransporter lipoprotein NalP (Autotransporter NalP) (NalP) (EC 3.4.21.-) [Cleaved into: NalP passenger domain; NalP translocator] | E6MVD9 | NALP | *Neisseria meningitidis* serogroup B / serotype 15 (strain H44/76) | Related bacteria |
| Arylamine N-acetyltransferase (NAT) (EC 2.3.1.5) | O86309 | NAT | *Mycolicibacterium smegmatis* (Mycobacterium smegmatis) | Related bacteria |
| Copper-containing nitrite reductase (EC 1.7.2.1) (Cu-NIR) | P25006 | NIR | *Achromobacter cycloclastes* | Related bacteria |
| Parathion hydrolase (EC 3.1.8.1) (Phosphotriesterase) (PTE) | P0A433 | OPD | *Sphingobium fuliginis* (strain ATCC 27551) | Related bacteria |
| Porin | P39767 | PORI | *Fuscovulum blasticum* (*Rhodobacter blasticus*) (*Rhodopseudomonas blastica*) | Related bacteria |
| Serine protease (EC 3.4.21.-) | Q9EXR9 | Q9EXR9 | *Bacillus intermedius* | Related bacteria |
| Multidrug resistance protein, putative | Q9KS51 | Q9KS51 | *Vibrio cholerae* serotype O1 (strain ATCC 39315 / El Tor Inaba N16961) | Related bacteria |
| Surface protein A | Q9RP17 | Q9RP17 | *Neisseria meningitidis* | Related bacteria |
| Large ribosomal subunit protein uL1 (50S ribosomal protein L1) | P27150 | RL1 | *Thermus thermophilus* | Related bacteria |
| Ribonuclease PH (RNase PH) (EC 2.7.7.56) (tRNA nucleotidyltransferase) | P50597 | RNPH | *Pseudomonas aeruginosa* (strain ATCC 15692 / DSM 22644 / CIP 104116 / JCM 14847 / LMG 12228 / 1C / PRS 101 / PAO1) | Related bacteria |
| Ribosome-recycling factor (RRF) (Ribosome-releasing factor) | P0A808 | RRF | *Shigella flexneri* | Related bacteria |
| Steroid Delta-isomerase (EC 5.3.3.1) (Delta(5)-3-ketosteroid isomerase) | P07445 | SDIS | *Pseudomonas putida* (*Arthrobacter siderocapsulatus*) | Related bacteria |
| Uncharacterized protein YhaI | O07517 | YHAI | *Bacillus subtilis* (strain 168) | Related bacteria |
| Cytochrome c peroxidase, mitochondrial (CCP) (EC 1.11.1.5) | P00431 | CCPR | *Saccharomyces cerevisiae* (strain ATCC 204508 / S288c) (Baker's yeast) | Related fungi |
| Cutinase 1 (EC 3.1.1.74) (Cutin hydrolase 1) | P00590 | CUTI1 | *Fusarium vanettenii* (*Neocosmospora pisi*) | Related fungi |
| Delta-aminolevulinic acid dehydratase (ALADH) (EC 4.2.1.24) (Porphobilinogen synthase) | P05373 | HEM2 | *Saccharomyces cerevisiae* (strain ATCC 204508 / S288c) (Baker's yeast) | Related fungi |
| Serine/threonine-protein kinase/endoribonuclease IRE1 (Endoplasmic reticulum-to-nucleus signaling 1) [Includes: Serine/threonine-protein kinase (EC 2.7.11.1); Endoribonuclease (EC 3.1.26.-)] | P32361 | IRE1 | *Saccharomyces cerevisiae* (strain ATCC 204508 / S288c) (Baker's yeast) | Related fungi |
| Lipase B (EC 3.1.1.3) (CALB) | P41365 | LIPB | *Pseudozyma antarctica* (Yeast) (*Candida antarctica*) | Related fungi |
| Glycogen phosphorylase (EC 2.4.1.1) | P06738 | PHSG | *Saccharomyces cerevisiae* (strain ATCC 204508 / S288c) (Baker's yeast) | Related fungi |
| Vanillyl-alcohol oxidase (EC 1.1.3.38) (4-allylphenol oxidase) (Aryl-alcohol oxidase) | P56216 | VAOX | *Penicillium simplicissimum* | Related fungi |

**Table S5.** Final candidate targets of Monascorubrin in *Escherichia coli*

| No. | Selected target | Gene name | UniProt ID |
| --- | --- | --- | --- |
| 1 | Multidrug efflux pump subunit AcrA | ACRA | P0AE06 |
| 2 | Multidrug efflux pump subunit AcrB | ACRB | P31224 |
| 3 | Aminoglycoside efflux pump AcrD | ACRD | P24177 |
| 4 | Multidrug efflux pump accessory protein AcrZ | ACRZ | P0AAW9 |
| 5 | N-acetylmuramoyl-L-alanine amidase AmiA | AMIA | P36548 |
| 6 | N-acetylmuramoyl-L-alanine amidase AmiB | AMIB | P26365 |
| 7 | N-acetylmuramoyl-L-alanine amidase AmiC | AMIC | P63883 |
| 8 | Beta-lactamase (EC 3.5.2.6) (Cephalosporinase) (CSase) | AMPC | P00811 |
| 9 | Beta-lactamase / Cephalosporinase | AMPC | P00811 |
| 10 | ATP synthase subunit c / F₀ sector subunit c | ATPE | P68699 |
| 11 | Pimeloyl-[acyl-carrier protein] methyl ester esterase / Carboxylesterase BioH | BIOH | P13001 |
| 12 | Vitamin B12 transporter BtuB (Cobalamin receptor) (Outer membrane cobalamin translocator) | BTUB | P06129 |
| 13 | Cytidine deaminase (EC 3.5.4.5) (Cytidine aminohydrolase) (CDA) | CDD | P0ABF6 |
| 14 | ATP-dependent Clp protease proteolytic subunit | CLPP | P0A6G7 |
| 15 | Nicotinate-nucleotide–dimethylbenzimidazole phosphoribosyltransferase | COBT | P36562 |
| 16 | Periplasmic serine endoprotease DegP | DEGP | P0C0V0 |
| 17 | Periplasmic pH-dependent serine endoprotease DegQ | DEGQ | P39099 |
| 18 | Serine endoprotease DegS | DEGS | P0AEE3 |
| 19 | DNA protection during starvation protein | DPS | P0ABT2 |
| 20 | Dr hemagglutinin structural subunit | DRAA | P24093 |
| 21 | Multidrug export protein EmrA | EMRA | P27303 |
| 22 | Multidrug export protein EmrB | EMRB | P0AEJ0 |
| 23 | Multidrug transporter EmrE | EMRE | P23895 |
| 24 | Probable multidrug resistance protein EmrK | EMRK | P52599 |
| 25 | Probable multidrug resistance protein EmrY | EMRY | P52600 |
| 26 | 3-hydroxydecanoyl-[ACP] dehydratase / FabA | FABA | P0A6Q3 |
| 27 | 3-oxoacyl-[acyl-carrier-protein] synthase 1 (EC 2.3.1.41) (3-oxoacyl-[acyl-carrier-protein] synthase I) (Beta-ketoacyl-ACP synthase I) (KAS I) | FABB | P0A953 |
| 28 | Enoyl-[acyl-carrier-protein] reductase [NADH] FabI (ENR) (EC 1.3.1.9) (NADH-dependent enoyl-ACP reductase) | FABI | P0AEK4 |
| 29 | 3-hydroxyacyl-[ACP] dehydratase FabZ | FABZ | P0A6Q6 |
| 30 | Iron(3+)-hydroxamate-binding protein FhuD (Ferric hydroxamate uptake protein D) (Ferrichrome-binding periplasmic protein) (Iron(III)-hydroxamate-binding protein FhuD) | FHUD | P07822 |
| 31 | Ribosome-recycling factor / Ribosome-releasing factor | FRR | P0A805 |
| 32 | Esterase FrsA | FRSA | P04335 |
| 33 | ATP-dependent zinc metalloprotease FtsH | FTSH | P0AAI3 |
| 34 | Glutamate mutase sigma subunit | GLMS | P58620 |
| 35 | Rhomboid protease GlpG / intramembrane serine protease | GLPG | P09391 |
| 36 | Co-chaperonin GroES / 10 kDa chaperonin | GROES | P0A6F9 |
| 37 | Protease HtpX | HTPX | P23894 |
| 38 | Signal peptidase I / Leader peptidase I | LEPB | P00803 |
| 39 | Metalloprotease LoiP | LOIP | P25894 |
| 40 | Macrolide export protein MacA | MACA | P75830 |
| 41 | Macrolide export ATP-binding/permease protein MacB | MACB | P75831 |
| 42 | Multidrug transporter MdfA | MDFA | P0AEY8 |
| 43 | Multidrug resistance protein MdtA | MDTA | P76397 |
| 44 | Multidrug resistance protein MdtB | MDTB | P76398 |
| 45 | Multidrug resistance protein MdtC | MDTC | P76399 |
| 46 | Multidrug resistance protein MdtE | MDTE | P37636 |
| 47 | Multidrug resistance protein MdtF | MDTF | P37637 |
| 48 | Multidrug resistance protein MdtK | MDTK | P37340 |
| 49 | Multidrug resistance protein MdtM | MDTM | P39386 |
| 50 | Multidrug resistance protein MdtN | MDTN | P32716 |
| 51 | Multidrug resistance protein MdtO | MDTO | P32715 |
| 52 | Oxygen-insensitive NAD(P)H nitroreductase (EC 1.-.-.-) (Dihydropteridine reductase) (EC 1.5.1.34) (FMN-dependent nitroreductase) | NFSB | P38489 |
| 53 | Arylamine N-acetyltransferase | NHOA | P77567 |
| 54 | NAD-capped RNA hydrolase NudC (DeNADding enzyme NudC) (EC 3.6.1.-) (NADH pyrophosphatase) (EC 3.6.1.22) | NUDC | P32664 |
| 55 | Outer membrane protein A (OmpA) (Outer membrane porin A) (Outer membrane protein 3A) (Outer membrane protein B) (Outer membrane protein II*) (Outer membrane protein d) | OMPA | P0A910 |
| 56 | Outer membrane protein A / OmpA | OMPA | P0A910 |
| 57 | Outer membrane porin C / OmpC | OMPC | P06996 |
| 58 | Outer membrane porin F (Outer membrane protein 1A) (Outer membrane protein B) (Outer membrane protein F) (Outer membrane protein IA) (Porin OmpF) | OMPF | P02931 |
| 59 | Outer membrane porin F / OmpF | OMPF | P02931 |
| 60 | Phospholipase A1 (EC 3.1.1.32) (EC 3.1.1.4) (Detergent-resistant phospholipase A) (DR-phospholipase A) (Outer membrane phospholipase A) (OM PLA) (OMPLA) (Phosphatidylcholine 1-acylhydrolase) | PA1 | P0A921 |
| 61 | Bifunctional chorismate mutase/prephenate dehydratase | PHEA | P0A9J8 |
| 62 | Outer membrane porin PhoE | PHOE | P02932 |
| 63 | Phosphotriesterase homology protein | PHP | P45548 |
| 64 | Glycerol-3-phosphate acyltransferase | PLSB | P0A7A7 |
| 65 | 1-acyl-sn-glycerol-3-phosphate acyltransferase | PLSC | P26647 |
| 66 | Tail-specific protease | PRC | P23865 |
| 67 | Truncated inactive ribonuclease PH / inactive RNase PH | RPH | P0CG19 |
| 68 | Large ribosomal subunit protein uL1 / 50S ribosomal protein L1 | RPLA | P0A7L0 |
| 69 | Regulator of sigma-E protease RseP / site-2 protease | RSEP | P0AEH1 |
| 70 | Probable protease SohB | SOHB | P0AG14 |
| 71 | Protease 4 / Signal peptide peptidase | SPPA | P08395 |
| 72 | Thioesterase 1/protease 1/lysophospholipase L1 (TAP) (Acyl-CoA thioesterase 1) (TESA) (EC 3.1.2.2) (Acyl-CoA thioesterase I) (Arylesterase) (EC 3.1.1.2) (Lysophospholipase L1) (EC 3.1.1.5) (Oleoyl-[acyl-carrier-protein] hydrolase) (EC 3.1.2.14) (Phospholipid degradation C) (Pldc) (Protease 1) (EC 3.4.21.-) (Protease I) (Thioesterase I/protease I) (TEP-I) | TESA | P0ADA1 |
| 73 | Outer membrane protein TolC / multidrug efflux channel | TOLC | P02930 |
| 74 | T-protein; chorismate mutase/prephenate dehydrogenase | TYRA | P07023 |
| 75 | Thymidylate synthase (TS) (TSase) (EC 2.1.1.45) | TYSY | P0A884 |
| 76 | Esterase YbfF | YBFF | P75736 |
| 77 | Metalloprotease YcaL | YCAL | P43674 |
| 78 | Esterase YjfP | YJFP | P39298 |
| 79 | Esterase YpfH | YPFH | P76561 |
| 80 | Cell division protein ZipA (FtsZ interacting protein A) | ZIPA | P77173 |

**Table S6.** Final candidate targets of Monascorubrin in *Staphylococcus aureus*

| No. | Selected target | Gene name | UniProt ID |
| --- | --- | --- | --- |
| 1 | Bifunctional autolysin | ATL | P0C5Z8 |
| 2 | ATP synthase subunit c / F₀ sector subunit c | ATPE | Q2G2F6 |
| 3 | Beta-lactamase | BLAZ | P00807 |
| 4 | 3-hydroxyacyl-[ACP] dehydratase FabZ | FABZ | Q2FWF5 |
| 5 | Ribosome-recycling factor / Ribosome-releasing factor | FRR | P68786 |
| 6 | Co-chaperonin GroES / 10 kDa chaperonin | GROES | P0A014 |
| 7 | Lipase 2 / Glycerol ester hydrolase 2 | LIP2 | P10335 |
| 8 | Autolysin / N-acetylmuramoyl-L-alanine amidase | LYTA | P24556 |
| 9 | Glycyl-glycine endopeptidase LytM / autolysin-related cell-wall peptidase | LYTM | O33599 |
| 10 | Multidrug export protein MepA | MEPA | Q2G140 |
| 11 | 6-phospho-N-acetylmuramidase | MUPG | A0A0H2XHV5 |
| 12 | Quinolone resistance protein NorB | NORB | Q2FYJ5 |
| 13 | Quaternary ammonium compound-resistance protein QacC / multidrug resistance protein | QACC | P14319 |
| 14 | Large ribosomal subunit protein uL1 / 50S ribosomal protein L1 | RPLA | Q2G0P0 |
| 15 | Putative zinc metalloprotease SA1105 | SA1105 | P63333 |
| 16 | Putative zinc metalloprotease SAV1262 | SAV1262 | P63332 |
| 17 | Multidrug efflux pump SdrM | SDRM | Q99S97 |
| 18 | Multidrug resistance efflux pump SepA | SEPA | Q99S98 |
| 19 | N-acetylmuramoyl-L-alanine amidase Sle1 | SLE1 | P0C1U7 |
| 20 | Staphylococcal protein A / Immunoglobulin G-binding protein A | SPA | P02976 |
| 21 | Glutamyl endopeptidase / Staphylococcal serine proteinase / V8 protease | SSPA | P0C1U8 |
| 22 | Tetracycline resistance efflux protein Tet(K) | TET | P02983 |

**Table S7.** Final candidate targets of Monascorubrin in *Candida albicans*

| No. | Selected target | Gene name | UniProt ID |
| --- | --- | --- | --- |
| 1 | Cytochrome c peroxidase, mitochondrial | CCP1 | Q5AEN1 |
| 2 | Lipase 1 | LIP1 | O94091 |
| 3 | Lipase 10 | LIP10 | Q9P4E5 |
| 4 | Lipase 2 | LIP2 | Q9P8W5 |
| 5 | Lipase 3 | LIP3 | Q9P8W2 |
| 6 | Lipase 4 | LIP4 | Q9P8W1 |
| 7 | Lipase 5 | LIP5 | Q9P8W0 |
| 8 | Lipase 6 | LIP6 | Q9P4E8 |
| 9 | Lipase 7 | LIP7 | Q9P4E7 |
| 10 | Lipase 8 | LIP8 | Q9P8V9 |
| 11 | Lipase 9 | LIP9 | Q9P4E6 |
